# The highly polymorphic *CYP6M7* cytochrome P450 gene partners with the directionally selected *CYP6P9a* and *CYP6P9b* genes to expand the pyrethroid resistance front in the malaria vector *Anopheles funestus* in Africa

**DOI:** 10.1186/1471-2164-15-817

**Published:** 2014-09-27

**Authors:** Jacob M Riveron, Sulaiman S Ibrahim, Emmanuel Chanda, Themba Mzilahowa, Nelson Cuamba, Helen Irving, Kayla G Barnes, Miranda Ndula, Charles S Wondji

**Affiliations:** Vector Biology Department, Liverpool School of Tropical Medicine, Pembroke place, Liverpool, L3 5QA UK; Ministry of Health, National Malaria Control Centre, Lusaka, Zambia; Malaria Alert Centre, College of Medicine, Blantyre, Malawi; National Institute of Health, Maputo, Mozambique

**Keywords:** *Anopheles funestus*, Malaria, Pyrethroids, Insecticide resistance, Cytochrome P450, *CYP6M7*, Directional selection

## Abstract

**Background:**

Pyrethroid resistance in the major malaria vector *Anopheles funestus* is rapidly expanding across Southern Africa. It remains unknown whether this resistance has a unique origin with the same molecular basis or is multifactorial. Knowledge of the origin, mechanisms and evolution of resistance are crucial to designing successful resistance management strategies.

**Results:**

Here, we established the resistance profile of a Zambian *An. funestus* population at the northern range of the resistance front. Similar to other Southern African populations, Zambian *An. funestus* mosquitoes are resistant to pyrethroids and carbamate, but in contrast to populations in Mozambique and Malawi, these insects are also DDT resistant. Genome-wide microarray-based transcriptional profiling and qRT-PCR revealed that the cytochrome P450 gene *CYP6M7* is responsible for extending pyrethroid resistance northwards. Indeed, *CYP6M7* is more over-expressed in Zambia [fold-change (FC) 37.7; 13.2 for qRT-PCR] than *CYP6P9a* (FC15.6; 8.9 for qRT-PCR) and *CYP6P9b* (FC11.9; 6.5 for qRT-PCR), whereas *CYP6P9a* and *CYP6P9b* are more highly over-expressed in Malawi and Mozambique. Transgenic expression of *CYP6M7* in *Drosophila melanogaster* coupled with *in vitro* assays using recombinant enzymes and assessments of kinetic properties demonstrated that *CYP6M7* is as efficient as *CYP6P9a* and *CYP6P9b* in conferring pyrethroid resistance. Polymorphism patterns demonstrate that these genes are under contrasting selection forces: the exceptionally diverse *CYP6M7* likely evolves neutrally, whereas *CYP6P9a* and *CYP6P9b* are directionally selected. The higher variability of *CYP6P9a* and *CYP6P9b* observed in Zambia supports their lesser role in resistance in this country.

**Conclusion:**

Pyrethroid resistance in Southern Africa probably has multiple origins under different evolutionary forces, which may necessitate the design of different resistance management strategies.

**Electronic supplementary material:**

The online version of this article (doi:10.1186/1471-2164-15-817) contains supplementary material, which is available to authorized users.

## Background

Malaria remains the most important vector-borne disease in the world. Nearly 216 million cases are reported each year, with an estimated 655,000 deaths in 2010, 86% of which occurred in children under the age of five [1]. Malaria control relies extensively on the use of insecticides, either in Long Lasting Insecticide Nets (LLINs) or as Indoor Residual Sprays (IRS). Unfortunately, increasing resistance to the available insecticide classes in the major malaria vectors, such as *Anopheles funestus*, is threatening the effectiveness of these control tools across Africa [2]. The recent WHO Global Plan for Insecticide Resistance Management [2] highlights the growing threat posed by insecticide resistance and calls for urgent action to implement suitable resistance management strategies against malaria vectors to ensure the continued effectiveness of control interventions. The elucidation of the molecular basis of insecticide resistance in malaria vectors is a crucial step in the design and implementation of these resistance management strategies.

Resistance to pyrethroid insecticides is of particular concern because these insecticides are the only class recommended by WHO for the impregnation of LLINs. Previous efforts to characterize the mechanisms of pyrethroid resistance in *An. funestus* have revealed that resistance is mainly driven by metabolic resistance with cytochrome P450 genes playing a major role while target-site resistance through knockdown resistance (*kdr*) was absent [3–6]. Two duplicated cytochrome P450 genes, *CYP6P9a* and *CYP6P9b*, were recently showed to play a key role in the resistance observed in the Southern African countries of Malawi and Mozambique [4]. The similarity in the gene expression profiles and the presence of a highly predominant resistance haplotype for each gene in the two countries suggested that the resistance had a unique origin [4]. However, recent susceptibility studies have demonstrated that the pyrethroid resistance front is expanding further northward across Southern Africa [5, 7, 8]. The factors behind this expansion remained uncharacterized, and it remains unknown if, beyond Malawi and Mozambique, the pyrethroid resistance front is still of a unique origin as previously suggested [4].

Resistance can spread from a single origin through gene flow between populations and can involve a single resistance mechanism. This phenomenon was responsible for the worldwide spread of DDT resistance under the control of the *CYP6G1* gene in the fruit fly [9]. The expansion of the resistance front could also originate from multiple independent selection events by different selection forces, potentially leading to the involvement of multiple resistance mechanisms in the overall resistance front. This phenomenon has been observed in the other major malaria vector *An. gambiae*, in which pyrethroid/DDT resistance is now widespread and is associated with various mechanisms, including two 1014 F/S knockdown resistance mutations [10, 11] and over-transcription of various metabolic genes [12–14]. It is not known if the expansion of pyrethroid resistance in Southern African populations of *An. funestus* is driven by different resistance mechanisms as in *An. gambiae* or by the same underlying resistance mechanism under the control of different genes. Furthermore, whether genes other than *CYP6P9a* and *CYP6P9b* are also responsible for the expansion of the resistance front remains to be determined. This information has important operational implications for the implementation of resistance management strategies against *An. funestus* across this region.

In addition, because the main tool used previously was a microarray chip based on a set of 8,540 *An. funestus* Expressed Sequenced Tags (ESTs) that did not cover the full transcriptome of this species [15], it is possible that other important resistance genes may contribute to the pyrethroid resistance previously characterized in Mozambique and Malawi. The availability of another RNAseq EST set with more than 15,000 ESTs [16] has enabled increased transcriptome coverage and a more comprehensive elucidation of the molecular basis of pyrethroid resistance in *An. funestus*.

The present study aimed to answer two main questions. i) Is the pyrethroid resistance front across Southern African *An. funestus* populations driven by same resistance mechanisms? ii) Are genes other than *CYP6P9a* and *CYP6P9b* also responsible for the previously reported resistance to pyrethroids in *An. funestus* in Mozambique and Malawi?

In this study, after establishing the resistance profile of a Zambian *An. funestus* population, we used genome-wide transcriptional and functional analyses to demonstrate that the cytochrome P450 *CYP6M7* has efficiently partnered with the previously detected *CYP6P9a* and *CYP6P9b* to expand the pyrethroid resistance front northward. Furthermore, we demonstrate that these three genes are under constrasting selection forces; the exceptionally polymorphic *CYP6M7* evolving neutrally, whereas *CYP6P9a* and *CYP6P9b* are both under directional selection.

## Methods

### Area of study and mosquito collection

Blood-fed female *An. funestus* adults resting indoors were collected in houses between 06.00 and 12.00 AM in Mbinga (Katete District) (14° 11' 0" S, 31° 52' 0" E) in eastern Zambia in October 2010. The Malawian sample was collected in the Chikwawa District (0° 45′ N, 34° 5′E) in Southern Malawi in July 2009 and April 2010. The Mozambican sample was collected in Tihuquine (Chokwe District) (24° 33' 37" S, 33° 1' 20" E) in southern Mozambique in January 2009 as recently described [4]. The collection method and rearing were conducted as described previously [5, 17]. F_1_ adults were generated from field-collected female mosquitoes and randomly mixed in cages for subsequent experiments.

### PCR species identification

All females used for individual oviposition were morphologically identified as belonging to the *An. funestus* group according to the key of [18]. A PCR assay was performed using the protocol of [19] to confirm that all females that laid eggs were *An. funestus s.s*.

### Bioassays

Insecticide susceptibility assays were performed using 2- to 5-day-old F_1_ adults from pooled families from Mbinga as described previously [5, 17] following the WHO protocol [20]. We tested the following insecticides: 0.75% permethrin (type I pyrethroid) and 0.05% deltamethrin (type II pyrethroid); 0.1% bendiocarb (carbamate); 5% malathion (organophosphate); and the organochlorines DDT (4%) and dieldrin (4%). The effect of the synergist PBO was assessed in combination with 0.75% permethrin, 4% DDT and 0.1% bendiocarb. For each insecticide, 100 female mosquitoes were pre-exposed to 4% PBO impregnated-paper for 1 h and immediately exposed to the insecticide paper for 1 h. Final mortality was assessed after 24 h and compared to the results obtained in the absence of PBO.

### Microarray

To detect the genes associated with the expansion of the pyrethroid resistance front in *An. funestus*, a new 8×60 k Agilent microarray chip was designed using the eArray program (Agilent) (A-MEXP-2374) by adding the 15,527 Expressed Sequence Tags (ESTs) generated from another transcriptome sequencing of *An. funestus* [16] to the previous 4×44 k array used by [4]. Each array contained 60mer probes designed from 8,540 ESTs generated from *An. funestus* transcriptome 454 sequencing [15] (2 probes for each EST), a set of 2850 *An. funestus* cDNAs from GenBank (2 probes for each EST), a set of P450 genes from the *rp1* and *rp2* QTL BAC sequence [21, 22] (3 probes for each gene), and the 13,000 transcripts of the complete *An. gambiae* genome. In addition, all of the *An. gambiae* detoxification genes previously present on the *An. gambiae* detox chip [23] were added to this chip with 3 probes for each gene to exploit possible gene sequence conservation between *An. gambiae* and *An. funestus*.

RNA was extracted from three batches of ten 2- to 5-day-old *An. funestus* females alive after exposure to 0.75% permethrin (Resistant, R) and unexposed mosquitoes of the fully susceptible laboratory strain FANG (Susceptible, S) using the Picopure RNA Isolation Kit (Arcturus). The quantity and quality of the extracted RNA were assessed using a NanoDrop ND1000 spectrophotometer (Thermo Fisher) and Bioanalyzer (Agilent, Santa Clara, CA, USA), respectively. Complementary RNA (cRNA) was amplified from each sample using the Agilent Quick Amp Labeling Kit (two-color) following the manufacturer’s protocol. cRNAs from the resistant samples (R) were labeled with cy5 dye, and cRNAs from the susceptible strain FANG (S) were labeled with the cy3 dye. cRNA quantity and quality were assessed before labeling using the NanoDrop and Bioanalyzer. Labeled cRNAs were hybridized to the arrays for 17 h at 65°C according to the manufacturer’s protocol. Five hybridizations were performed for each location by swapping the biological replicates.

Microarray data were analyzed using Genespring GX 12.0 software. To identify differentially expressed genes, a cut-off of 2-fold-change (FC) and a statistical significance of P < 0.01 with Benjamini-Hochberg correction for multiple testing and q < 0.01 with Storey with bootstrapping were applied. The predicted functions of all the transcripts and ESTs used for this microarray chip were identified by the BLAST2GO program [24, 25].

### Quantitative reverse transcriptase PCR

The expression patterns of some of the genes most associated with resistance based on the microarray analyses were assessed by qRT-PCR using the primers listed in Additional file 1: Table S1. In the case of the new candidate resistance gene *CYP6M7*, fold change was obtained from three independent primer pairs to further validate the microarray over-expression. One microgram of total RNA from each of the three biological replicates from the Resistant (R), Control (C) (mosquitoes from each location not exposed to insecticide) and FANG (S) populations for the three countries was used as the template for cDNA synthesis using Superscript III (Invitrogen) with oligo-dT20 and RNase H according to the manufacturer’s instructions. A serial dilution of cDNA was used to establish standard curves for each gene to assess PCR efficiency and quantitative differences between samples. qRT-PCR amplification was performed as described previously [4, 26]. The relative expression level and FC of each target gene in R and C relative to S were calculated according to the 2^-ΔΔCT^ method incorporating the PCR efficiency [27] after normalization with the housekeeping genes ribosomal protein S7 (*RSP7*; AGAP010592) and actin 5C (AGAP000651).

### Tissue-specific expression of candidate resistance genes

To identify the tissues where the main resistance genes are expressed, we performed a qRT-PCR assay for these genes in sets of cDNA synthesized from RNA extracted from the heads, thoraces and abdomens of female mosquitoes from Zambia that were not exposed to insecticides. A total of 30 females were dissected, and three sets of 10 heads, 10 abdomens and 10 thoraces were used for RNA extraction. The results were analyzed as described above, with the expression level of each gene in each body part determined in relation to the FC in the whole mosquito.

### Transgenic expression of candidate genes in *Drosophila*strains

To establish whether over-expression of the candidate gene *CYP6M7* alone can confer resistance to different pyrethroids, transgenic *Drosophila melanogaster* flies expressing this gene were generated using the GAL4/UAS system. The construction of the transgenic strain followed the protocol recently successfully used for the P450s *CYP6P9a* and *CYP6P9b* [4].

Briefly, full-length *CYP6M7* was amplified from cDNA using the Phusion High-Fidelity DNA Polymerase (Thermo Scientific) and cloned into the pJET1.2/blunt cloning vector (Thermo Scientific). The primers used are listed in Additional file 1: Table S1. After sequence analysis, one clone that was predominant in the three countries was selected to construct transgenic flies and cloned into the pUASattB vector using primers containing restriction sites for *EcoR*I and *Xba*I. Using the PhiC31 system, clones were transformed into the germ line of a *D. melanogaster* strain carrying the attP40 docking site on chromosome 2 ["y^1^w^67c23^; P attP40", "1;2"] by Genetic Services (MA, USA) to generate the transgenic line UAS-CYP6M7. Ubiquitous expression of the transgene *CYP6M7* in adult F_1_ progeny (experimental group) was obtained after crossing virgin females from the driver strain Act5C-GAL4 ["y [1] w[*]; P(Act5C-GAL4-w)E1/CyO","1;2"] (Bloomington Stock Center, IN, USA) with homozygote UAS-CYP6M7 males. Similarly, adult F_1_ control progeny (control group) with the same genetic background as the experimental group but without expression of *CYP6M7* were obtained by crossing virgin females from the driver strain Act5C-GAL4 and UAS recipient line males (which do not carry the pUASattb-CYP6M7 insertion).

**Insecticide contact bioassays**: Experimental and control F_1_*Drosophila melanogaster* females were selected for use in insecticide bioassays. A comparison of the mortality rates of the experimental group and control group was used to assess whether *CYP6M7* could confer resistance. Post-eclosion females that were 2 to 5 days old were used in a contact assay with the pyrethroids deltamethrin and permethrin. After assessing a range of doses to identify the best discriminating dose for flies, 2% permethrin- and 0.15% deltamethrin-impregnated filter papers were prepared in acetone and Dow Corning 556 Silicone Fluid (BHD/Merck, Germany) for bioassays. These discriminating doses differ from that of mosquitoes due to various differences between both species notably in size and cuticle thickness. These papers were rolled and introduced into 45 cc plastic vials to cover the entire wall. The vials were plugged with cotton soaked in 5% sucrose. Then, 20–25 flies were placed in each vial, and the mortality plus knockdown was scored after 1 h, 2 h, 3 h, 6 h, 12 h and 24 h of exposure to the insecticide. For all assays, at least 6 replicates were performed. Student’s t-test was used to compare the mortality plus knockdown of the experimental group with the control group.

**Confirmation of transgene expression in transgenic flies by qRT**-**PCR**: To confirm the expression of *CYP6M7* in the experimental group and the absence of expression in the control groups, total RNA was extracted from three pools of 5 flies. cDNA was synthesized as described above. The relative expression levels of the transgene were assessed by qRT-PCR in the experimental F_1_ progeny as well as in the respective controls with normalization with the *RPL11* housekeeping gene.

### Heterologous expression of candidate genes in *E. coli*

**Cloning of*CYP6M7***, ***CYP6P9a*and*CYP6P9b*for expression in*E. coli***: *The CYP6M7*, *CYP6P9a* and *CYP6P9b* genes were fused to a bacterial ompA + 2 leader sequence and expressed in *E. coli* JM109 cells using the pCW-ori + vector as previously described [28–30]. Briefly, a DNA fragment containing the coding sequence for the ompA signal peptide with a downstream alanine-proline linker and approximately the first 20 nucleotides of each gene was first amplified using KAPA HiFi PCR (Kappa Biosystems) and 50 ng of *E. coli* JM109 DNA as the template using the specific primers (OMPA + 2_F and the reverse primers: OMPA + 2-CYP6M7, OMPA + 2-CYP6P9a and OMPA + 2-CYP6P9b, Additional file 1: Table S1). The KAPA HiFi PCR conditions were: 1 cycle at 95°C for 5 min; 35 cycles of 94°C for 20 s, 57°C for 30 s and elongation at 72°C for 90 s; and 1 cycle at 72°C for 5 min.

Next, the same *CYP6M7* clone used for the *Drosophila* transgenic study, the *CYP6P9a* and *CYP6P9b* clones used previously [4], and the ompA + 2 PCR fragment were used as templates in a fusion PCR under the same conditions described above. The full-length sequence of each gene incorporating the ompA + 2 leader was ligated into a modified pCW-ori + vector plasmid, pB13 [29], via *EcoR*I and *XBa*I sites to produce pB13::ompA + 2-CYP6PM7, pB13::ompA + 2-CYP6P9a and pB13::ompA + 2-CYP6P9b. These constructs were sequenced to confirm the absence of PCR errors.

**Membrane preparation**: For all genes, *E. coli* JM109 cells were co-transformed with pB13::ompA + 2-CYP6M7, pB13::ompA + 2-CYP6P9a or pB13::ompA + 2-CYP6P9b and a plasmid containing the *An. gambiae* cytochrome P450 reductase, pACYC-AgCPR [28]. The expression of each gene, membrane isolation and P450 content were determined as previously described [28, 30]. The membranes were stored in aliquots at -80°C and assayed for total protein concentration using NanoDrop spectrophotometer, P450 concentration [31] and CPR activity by monitoring cytochrome c reduction [32]. The histidine-tagged *An. gambiae* cytochrome b5 (Cyt-b5) was generated as previously described by Stevenson et al. [30] and used for the metabolism assays.

**Metabolism assays**: Pyrethroids were dissolved in methanol (HPLC grade, Fisher Scientific) immediately before use at a final working concentration of 0.02 mM. As previously described by Stevenson et al. [30], the reactions consisted of the following: 45 pmoles of P450, 0.2 M Tris HCl pH 7.4, 0.25 mM MgCl_2_, 1 mM glucose-6-phosphate, 0.1 mM NADP + (Melford), 1 unit/ml glucose-6-phosphate dehydrogenase (G6PDH), 0.8 μM Cyt-b5 and 0.2 mM pyrethroid insecticides in a final volume of 200 ml. NADP + was excluded from the minus NADPH control. Membranes expressing P450 and CPR with Cyt-b5 were applied to the side of a 1.5 ml Eppendorf tube, and buffer was added to the bottom of the tube. After a 5 min pre-incubation, the reactions were initiated by vortexing and incubated at 30°C with shaking at 1000 rpm.

**HPLC analysis**: Reactions were stopped with 0.1 ml of cold methanol and incubated with shaking (1000 rpm) for 5 min at 30°C to dissolve all available pyrethroids. The samples were then centrifuged at 16,000 rpm for 10 min at 4°C, and 150 μl of the supernatant was transferred into HPLC vials. The quantity of pyrethroid remaining in the samples was determined by reverse-phase HPLC with a monitoring absorbance wavelength of 226 nm (Agilent 1260 Infinity). A 100 μl sample was loaded into an isocratic mobile phase of 90% methanol and 10% water with a flow-rate of 1 ml/min, and substrate peaks were separated on a 250 mm C18 column (Acclaim™ 120, Dionex) at 23°C.

**Turnover and kinetic assays**: To determine the turnover of P450s with pyrethroids, experiments with deltamethrin and permethrin were performed in which the incubation time was varied from 0 to 30 minutes. The turnover was calculated from the plot of the initial velocity vs. time at zero-order phase by fitting the data to a non-linear regression using the enzyme kinetic module of GraphPad Prism 6.03 (GraphPad Software Inc., La Jolla, CA, USA). Steady-state kinetic parameters were obtained by measuring the rate of reaction under linear conditions for 10 minutes while varying the substrate concentration from 0 to 20 μM. K_M_ and Vmax were established from the plot of substrate concentrations against the initial velocities and fitting of the data to the Michaelis-Menten equation using GraphPad Prism. Catalytic constants and efficiencies were determined from the steady-state parameters.

### Genetic variability of candidate genes

The full-length coding regions of *CYP6M7* were amplified from cDNA of permethrin-resistant samples from Malawi, Mozambique and Zambia and from the susceptible FANG strain to assess the role of allelic variation in resistance. The amplification was performed using the same cDNA synthesized for qRT-PCR with the Phusion polymerase, which was cloned and sequenced as described above.

In addition, to assess the potential association between allelic variation and pyrethroid resistance, a genomic fragment spanning the full-length *CYP6M7* gene and a portion of the 5’UTR region were amplified and directly sequenced in five susceptible (dead after a 1 h exposure) and five resistant (alive after a 1 h exposure to 0.75% permethrin) mosquitoes from Zambia, Malawi and Mozambique. The primers used are listed in Additional file 1: Table S1. Similarly, both *CYP6P9a* and *CYP6P9b* were also amplified and sequenced from the Zambia samples for comparison with the polymorphism profiles previously obtained in Malawi and Mozambique samples [4]. Polymorphic positions were detected through manual analysis of sequence traces using BioEdit and as sequence differences in multiple alignments using ClustalW [33]. DnaSP 5.1 [34] was used to define the haplotype phase (through the Phase program) and to assess genetic parameters of each gene such as nucleotide diversity π and haplotype diversity. A maximum likelihood phylogenetic tree of the haplotypes for each gene was constructed using MEGA 5.2 [35], and a haplotype network was built using the TCS program [36] (95% connection limit; gaps treated as a fifth state) to assess the potential connection between haplotypes and resistance phenotypes.

**Test of selection on*CYP6M7***: To assess the possibility of selection acting on *CYP6M7* in all three countries and its role in pyrethroid resistance, the departure from neutrality was tested using the codon-based Z test of selection. As implemented in MEGA5.2, this test uses the Nei and Gojobori method to compute the numbers of synonymous (dS) and non-synonymous (dN) substitutions per site and the numbers of potentially synonymous and potentially non-synonymous sites [37]. The dN/dS ratio was calculated, and the probability of rejecting the null hypothesis of strict neutrality (H0: dN = dS) in favor of the alternative purifying (H1: dN < dS) or positive selection hypotheses (H1: dN > dS) was estimated using the bootstrap method (1000 replicates) in MEGA5.2.

## Results

### Susceptibility status of *An. funestus*in Zambia

**Figure 1:**
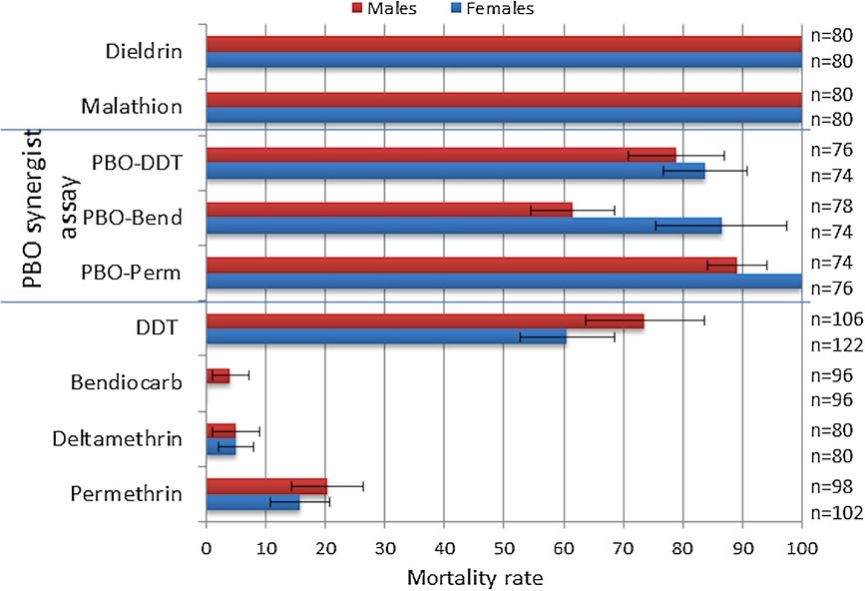
**Susceptibility profile of the** ***An*** *.* ***funestus*** **population from the Katete district in Zambia to the main insecticides and when exposed to the synergist piperonyl butoxide (PBO).** The data are presented as the mean of at least four replicates and error bars represent standard deviation.

The bioassays performed on the *An. funestus* population from Mbinga (Katete district) in Zambia indicated that this population is highly resistant to both type I (permethrin) and type II (deltamethrin) pyrethroids, with mortality rates of 15.7 and 5%, respectively, for females (Figure 1). This resistance level is higher than that previously reported in Malawi (47.2 and 42.3% mortality, respectively, for permethrin and deltamethrin) [5] but lower than that in Mozambique (no mortality after a 2 h exposure for both pyrethroid types) [17].

This Zambian population is also highly resistant to carbamates, with no mortality in females and only 5% mortality in males after a 1 h exposure to 0.1% bendiocarb. This level of carbamate resistance is much higher than that previously reported in Mozambican (70% mortality) [17] and Malawian (60% mortality) [5, 8] populations. Surprisingly, DDT resistance was also detected in the Zambian *An. funestus* population, with 60.6% mortality in females. This profile is significantly different from that in other Southern African populations; full susceptibility to DDT was observed in the Mozambican population, and only moderate resistance was observed in the Malawian population (93% mortality). Thus, despite their similar pyrethroid resistance profiles, the Zambian population differs from the Malawian and Mozambican populations. However, full susceptibility to malathion (organophosphate) and dieldrin was observed, with 100% mortality similar to that observed in the Mozambican and Malawian populations.

A near full susceptibility to permethrin was restored in the Zambian population after exposure to the synergist piperonyl butoxide (PBO), with 100% mortality in females. This result suggests that, as observed previously in Malawi and Mozambique, cytochrome P450 genes play a major role in the pyrethroid resistance observed in Zambia. Similarly, after PBO exposure, significant recovery of susceptibility to the carbamate bendiocarb from 0% to 86.5% was observed, further supporting an important role of P450s in the carbamate resistance observed in Zambia. However, the absence of a complete recovery of susceptibility suggests the potential involvement of other mechanisms. Exposure to PBO only moderately increased DDT mortality from 60.6 to 83.8%, suggesting that the potential role of P450s in DDT resistance might not be as important as in pyrethroid or carbamate resistance.

### Genome-wide microarray-based transcriptional profiling

**Figure 2:**
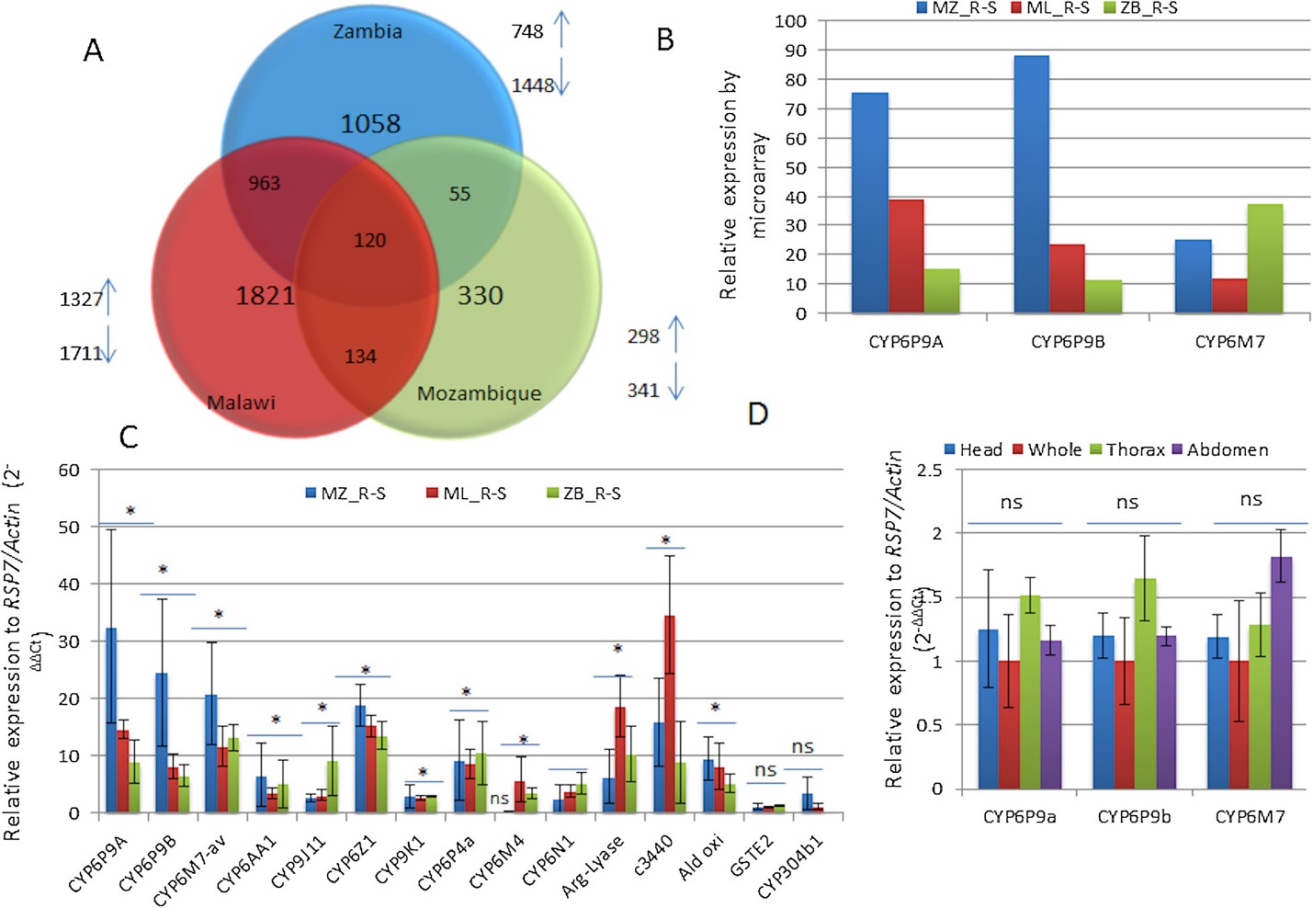
**Transcriptional profiling of resistant populations. A)** Summary of probes differentially regulated in each of the 3 countries. The Venn diagrams show the number of probes significantly (P < 0.01) up- or down-regulated (FC > 2) in each country as well as the commonly expressed probes. Upward arrows indicate up-regulated probes, and downward arrows represent down-regulated probes. **B)** Relative expression of the three main detoxification genes (*CYP6P9a, CYP6P9b* and *CYP6M7*) by microarray between the three countries (based on probes with highest expression); **C)** Differential expression of 15 genes up-regulated between permethrin-resistant (R) and -susceptible FANG (S) mosquitoes in the Mozambican (MZ), Malawian (ML) and Zambian field populations (ZB). Fold-change for *CYP6M7* was obtained from the average of three independent primer pairs. Error bars represent standard deviation (N = 3). The presence of * on top of the three fold changes for each gene indicates a statistically significant over-expression in all locations compared to the FANG susceptible strain. "ns" is added when the difference was not significant. **D)** Tissue-specific expression of *CYP6P9a*, *CYP6P9b* and *CYP6M7* in field permethrin-resistant female *An. funestus* mosquitoes.

A new genome-wide *An. funestus* custom Agilent microarray chip containing 60,000 probes (60mer) was used to identify genes associated with pyrethroid resistance in a Zambian population for the first time. The same chip was also used to detect novel resistance genes in Malawian and Mozambican populations that might have been missed by the previously less comprehensive chip [4]. Labeled complementary RNA (cRNA) was successfully obtained from 3 biological replicates of resistant (R) (mosquitoes alive after a 1 h exposure to 0.75% permethrin) from each country and susceptible (S) (unexposed mosquitoes from the fully susceptible laboratory strain FANG) populations. The number of probes that were differentially expressed (>2-fold change, FC) between R and S mosquitoes for each country and between them is indicated in Figure 2A (P < 0.01) and Additional file 2: Figure S1A (P < 0.05). Overall, 639 probes were differentially expressed in the Mozambican population (298 over-expressed and 341 under-expressed), 3038 in the Malawian population (1327 over-expressed and 1711 under-expressed) and 2196 in the Zambian population (748 over-expressed and 1448 under-expressed). A total of 120 probes were differentially expressed in all three countries.

**Genes commonly up**-**regulated in all three countries**: An analysis of the list of probes commonly up-regulated in mosquitoes from all three countries revealed that the two duplicated P450 genes *CYP6P9a* and *CYP6P9b* were among the most up-regulated genes for Mozambican and Malawian mosquitoes, with FCs of 75.5 and 39.4, respectively, for *CYP6P9a* and 88.2 and 24, respectively, for *CYP6P9b* (Additional file 1: Table S2). However, although these two genes were also significantly up-regulated in the Zambian sample, the FC was much lower than for the Mozambican and Malawian samples, with FCs of only 15.6 and 11.9, respectively, for *CYP6P9a* and *CYP6P9b*. This two to seven-fold decrease in the expression level of both genes from south Mozambique to Malawi and to Zambia was observed across the three distinct microarray probes used for each gene (Figure 2B). Such change in expression level suggests a variation in the roles these duplicated P450 genes play in the pyrethroid resistance observed across Southern Africa.

This hypothesis was further supported by the observation that the most highly over-expressed detoxification gene in the Zambian sample was another CYP6 family P450 gene, *CYP6M7*, which displayed an FC of 37.7, corresponding to a 2- to 3-fold greater up-regulation compared to *CYP6P9a* and *CYP6P9b*. This gene is located in the genomic region spanning the pyrethroid resistance *rp2* QTL on chromosome 2 L detected in the FUMOZ-R pyrethroid-resistant laboratory strain [21]. *CYP6M7* is the ortholog of the *CYP6M3* gene in *An. gambiae* (AGAP008213), which is over-expressed in resistant mosquitoes but with a much lower FC than that described in this study [26, 38]. *CYP6M7* was also up-regulated in the Mozambican and Malawian samples but with a lower FC than in the Zambia sample (25.7 and 12.5, respectively). This result suggests that *CYP6M7* might play a more important role in the pyrethroid resistance observed in Zambia than the duplicated *CYP6P9a* and *CYP6P9b* genes.

Another novel gene identified using the 8x60k chip was the P450 *CYP6AA1*, which was also commonly up-regulated in the samples from all three countries, with two-fold over-expression in the Mozambican sample (FC = 13.2) than in the Malawian (FC = 5.2) and Zambian (FC = 5.3) samples. This gene is the ortholog of *CYP6AA3* in *An. minimus*, which was recently shown to metabolize pyrethroids [39] and is located on the 2R chromosome in the *rp1* QTL in the same P450 cluster with *CYP6P9a* and *CYP6P9b* [22]. Other newly identified detoxification genes that were up-regulated in the samples from all three countries include three other P450s, *CYP6Y2* (located in *rp2* QTL), *CYP304B1* and *CYP9K1*, although these genes were expressed at lower levels (FC < 4, see Additional file 1: Table S2).

Two other P450 genes, *CYP9J11* (located around the *rp3* QTL) and *CYP6P2* (located around *rp1*), were also up-regulated in the three populations (Additional file 1: Table S2). Other genes commonly up-regulated in the three countries are listed in Additional file 1: Table S2.

**Probes commonly down**-**regulated in all three countries**: The most down-regulated genes in the samples from all three countries were previously reported to be down-regulated in Mozambican and Malawian samples [4] and include a cationic amino acid transporter, a monkey king protein gene, an isoform c gene and several probes for cytochrome c oxidase subunit 3 (Additional file 1: Table S3).

### Transcriptional profiling of the Zambian population

The transcriptional profile of the Zambian population was analyzed further because it had not been previously performed with the previous 4×44 k chip. This analysis revealed that, in addition to the main genes *CYP6P9a*, *CYP6P9b* and *CYP6M7*, other detoxification genes were up-regulated. The majority of these genes encode cytochrome P450s; nineteen P450 genes were up-regulated (P < 0.01) (Table 1). These P450s include *CYP6M4* (FC = 5.1) which was also up-regulated in the Malawian sample (FC = 4.3) (Table 1) but not the Mozambican sample (Table 1), *CYP315A1* (FC = 4.5) and *CYP6N1* (FC = 2.9).

**Table 1:** **The most up-regulated detoxification genes in Zambia (ZB), Malawi (ML) and Mozambique (MZ) (P < 0.01)**

Systematic name	Orthologs in ***An. gambiae***	Fold change (FC)			Description
		ZB	ML	MZ	
Afun007663 (CYP6M7)	AGAP008213-PA	37.7	12.5	25.7	Cytochrome p450
CYP6P9a	AGAP002865-PA	15.6	39.4	75.5	Cytochrome p450
CYP6P9b	AGAP002865-PA	11.9	24.0	88.2	Cytochrome p450
Afun008614 (CYP6AA1)	AGAP002862-PA	5.3	5.2	13.2	Cytochrome p450
Afun007346	AGAP007990-PA	2.7	3.1	3.5	Glucosyl glucuronosyl transferases
Afun007469 (CYP9J11)	AGAP012296-PA	4.1	4.8	4.0	Cytochrome p450
CYP6Y2		3.9	2.9	4.4	Cytochrome p450
Afun012197 (CYP6Z1)	AGAP003066-PA	2.8	2.9	3.9	Cytochrome p450
Afun009335 (CYP6AG1)	AGAP003343-PA	2.4	2.7	2.6	Cytochrome p450
Afun012194 (CYP6P2)	AGAP002869-PA	2.6	2.7	3.9	Cytochrome p450
Afun007769 (CYP9K1)	AGAP000818-PA	3.6	2.4	2.3	Cytochrome p450
Afun000493	AGAP006225-PA	3.0	2.2	2.6	Aldehyde oxidase
Afun010360	AGAP006222-PA	2.8	2.0	2.1	Glucosyl glucuronosyl transferases
combined_c920 (GSTe2)		2.5	3.4	2.8	Glutathione-s-transferase gst
Afun009227	AGAP008141-PA	25.4	66.3		Argininosuccinate lyase
Afun010614	AGAP006380-PA	4.0	2.5		atp-binding cassette sub-family a member
Afun007482	AGAP002693-PA	3.9	2.3		atp-binding cassette sub-family f member 2
Afun011877	AGAP013384-PA	2.5	2.1		atp-binding cassette transporter
Afun002473	AGAP000553-PA	6.6	4.5		atp-binding-cassette protein
Afun009492	AGAP001722-PA	12.0	5.9		Carboxylesterase
CD577459.1		2.3	3.0		Cuticle protein
CYP6M4		5.1	5.3		Cytochrome p450
Afun009522	AGAP012292-PA	2.4	4.5		Cytochrome p450
Afun003394 (CYP315A1)	AGAP000284-PA	4.5	2.4		Cytochrome p450
Afun007127 (CYP4C36)	AGAP009241-PA	2.1	2.4		Cytochrome p450
Afun007499 (GSTd1-5)	AGAP004164-PA	2.8	2.9		Glutathione transferase
combined_c4173		4.6	4.8		Glycoprotein 93
combined_c557		7.6	11.8		Trypsin
CD578169.1		3.0	6.0		Trypsin
Afun007646	AGAP006225-PA		2.0	3.4	Aldehyde oxidase
Afun008347	AGAP009828-PA		4.2	2.2	Chymotrypsin 1
Afun013935	AGAP010917-PA	2.2			Carboxylesterase
Afun013921	AGAP006709-PA	31.3			Chymotrypsin 1
Afun004392	AGAP008213-PA	2.3			Cytochrome p450
Afun008909	AGAP002416-PA	2.1			Cytochrome p450
Afun012666	AGAP002429-PA	2.1			Cytochrome p450
Afun008354 (GSTD3)	AGAP004382-PA	7.8			Glutathione transferase
Afun013481(GSTe1)	AGAP009195-PA	2.3			Glutathione-s-transferase gst
Afun011899	AGAP012514-PA	2.0			Short-chain dehydrogenase
combined_c4812		2.7			Short-chain dehydrogenase
AGAP007662-RA	AGAP007662-RA	2.4			Short-chain dehydrogenase

Two carboxylesterases were also up-regulated in Zambia. The *COEAE1A* gene (FC = 12), an ortholog of AGAP001722 in *An. gambiae* and *COE09916*, an ortholog of AGAP010917 in *An. gambiae* (FC = 2.2). *COEAE1A* was also over-expressed in the Malawian sample (FC = 6.1) but not the Mozambican sample, whereas *COE09916* was only over-expressed in the Zambian sample.

Another important detoxification gene family that was up-regulated in the Zambian sample is the glutathione S-transferases, with 4 genes notably over-expressed (Table 1). *GSTD3* was the most highly over-expressed member of this family (FC = 7.8) whereas the three other GSTs were up-regulated at a lower level (FC < 3); *GSTD1*-*5*, *GSTe2* and *GSTe1. GSTD3* is not over-expressed in the DDT-susceptible populations of Mozambique and Malawi.

Other up-regulated gene families include aldehyde oxidases, ABC transporters, proteases (such as trypsin and chymotrypsin 1), cuticle proteins, UDP glucosyl transferases, heat shock proteins (*HSP70*) argininosuccinate lyase and short chain dehydrogenases (Table 1).

### Validation of the microarray results by qRT-PCR

Quantitative real-time PCR (qRT-PCR) was used to validate the microarray results for fifteen of the most up-regulated detoxification genes in the samples from the three countries, including 11 P450 genes, one glutathione S-transferase gene (*GSTe2*), the argininosuccinate lyase gene, one aldehyde oxidase gene and one gene with unknown function (Combined_c3440). The qRT-PCR results confirmed the over-expression patterns observed by microarray, although with generally lower fold change values (Figure 2B). A similar expression pattern was observed for the control samples that were not exposed to insecticides from each of the three countries (Additional file 2: Figure S1B). Particular attention was paid to the *CYP6M7* to validate its high over-expression. The qRT-PCR results from the three independent primer pairs on *CYP6M7* confirmed that this gene is over-expressed in the three countries with FC of 21.0, 11.75 and 13.25 respectively in Mozambique, Malawi and Zambia when averaging over the three primers. In Zambia, the *CYP6M7* was most over-expressed (FC 13.25) than *CYP6P9a* (FC 8.9) and *CYP6P9b* (FC 6.53) although the difference is significant only against *CYP6P9b*. The Mozambique sample exhibited the highest over-expression for the three main pyrethroid candidate resistance gene in correlation with microarray in line with the high pyrethroid resistance observed in this population [17]. However, in Malawi, no significant difference was observed in the expression of the three genes in contrast to microarray. A notable higher over-expression was observed for the *CYP6Z1* P450 gene than with microarray for all the three countries (13 < FC < 18.9 for qRT-PCR while FC < 3 for microarray). A significant correlation between the qRT-PCR and microarray results was observed in the combined sample of the three countries (R^2^ = 0.4;P<0.05) (Additional file 2: Figure S1C).

**Tissue**-**specific expression profile of main candidate resistance genes**: Assessment of the tissue-specific expression profile of the main resistance genes by qRT-PCR showed that *CYP6P9a*, *CYP6P9b* and *CYP6M7* were all similarly expressed in the head, thorax and abdomen (Figure 2C). Indeed, the fold-change observed in these body parts is not significantly different from that of the whole body. This result suggests that expression of these genes is likely to be ubiquitous rather than specific to tissues involved in detoxification, such as the midgut, Malpighian tubules, or fat body [40].

### Transgenic expression of *CYP6M7*in *Drosophila*flies

**Figure 3:**
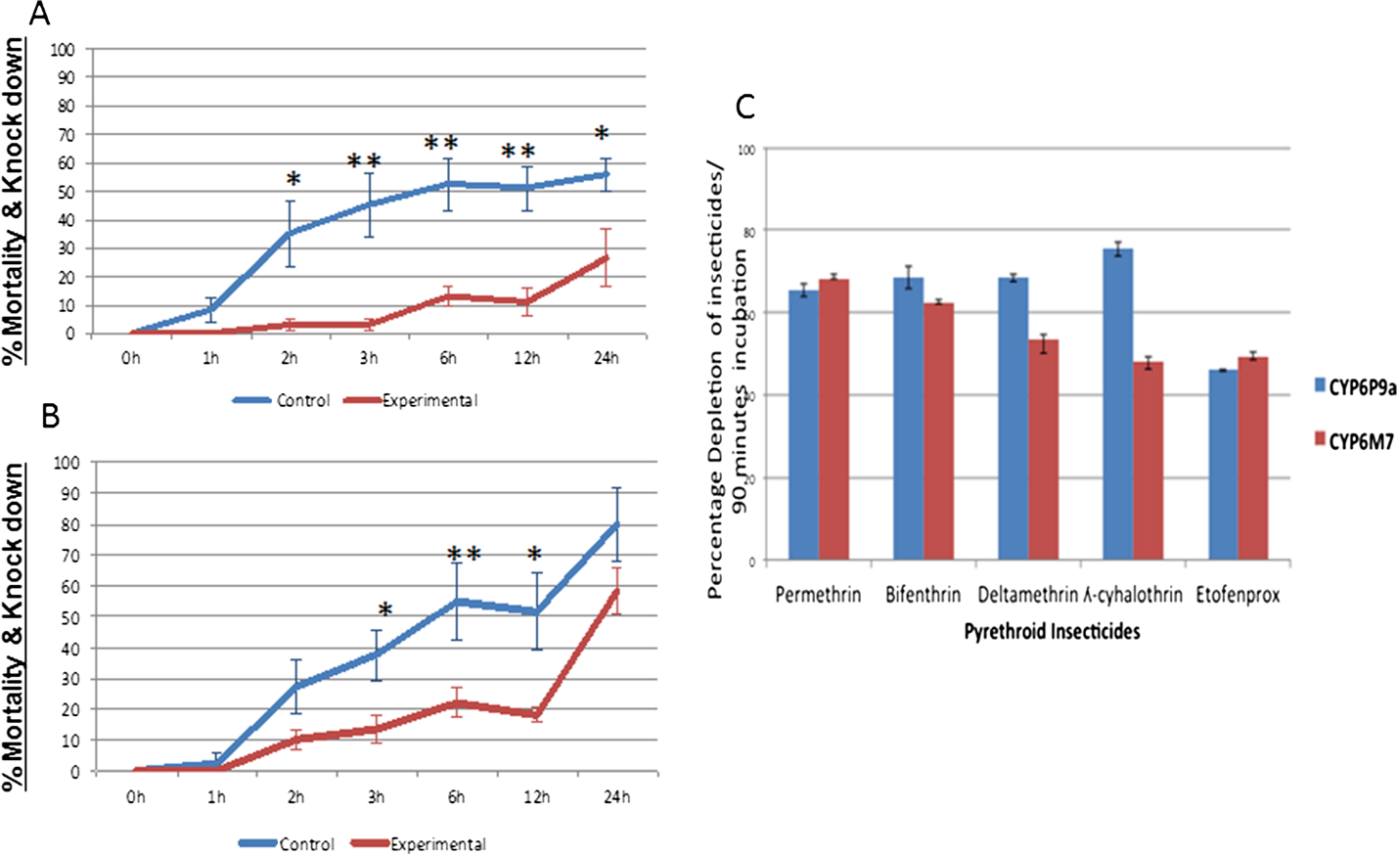
**Functional confirmation of the role of** ***CYP6M7*** **in pyrethroid resistance. A)** Transgenic expression of *CYP6M7* in *Drosophila*. Results of a bioassay with 2% permethrin **(A)** and 0.15% deltamethrin **(B)** against the transgenic Act5C-CYP6M7 strain (Experimental) and the progeny from the cross between the UAS-CYP6M7 females and w^1118^ males (which do not over-express the P450 transgene) (Control). The data shown are the mean ± SEM (n = 6). **(C)** The proportion of 10 μM insecticide cleared by 0.1 μM P450 with 0.8 μM cyt b5 in the presence of NADPH is indicated by bar height. Error bars represent standard deviation (N = 3).

To confirm that the over-transcription of *CYP6M7* alone can confer pyrethroid resistance, transgenic *D. melanogaster* individuals expressing a *CYP6M7* allele (derived from permethrin resistant field mosquitoes) under the control of the ubiquitous Act5C-GAL4 driver were successfully generated. qRT-PCR analysis confirmed that *CYP6M7* was expressed only in transgenic Act5C-CYP6M7 F_1_ progeny (2^-ΔΔCT^ of 0.17); virtually no expression was observed in the control (same genetic background but without *CYP6M7* gene) (2^-ΔΔCT^ of 4.8 × 10^-5^) (Additional file 2: Figure S1D). Contact bioassays performed with 2% permethrin (type I pyrethroid) and 0.15% deltamethrin (type II) revealed that flies over-expressing *CYP6M7* were resistant to both pyrethroid types, resulting in a significantly reduced mortality/knockdown rate for both insecticides compared to that observed for control flies. For permethrin, significantly reduced mortality/knockdown rates were recorded at five different exposure times of transgenic Act5C-CYP6M7 individuals when compared with the control group not expressing *CYP6M7*. These exposure times were 2 h (3.15 vs. 35.04%, P = 0.027), 3 h (3.15 vs. 44.92%, P = 0.0066), 6 h (13.20 vs. 52.39%, P = 0.0039), 12 h (11 vs. 50.96%, P = 0.0023) and 24 h (26.57 vs. 55.59%, P = 0.038) (Figure 3A). Similarly, a significant reduction in mortality/knockdown rates was also recorded for deltamethrin in the transgenic Act5C-CYP6M7 flies compared to the control group after 3 h (13.52 vs. 37.5%, P = 0.037), 6 h (22.10 vs. 54.71%, P = 0.041) and 12 h (18.1 vs. 51.30%, P = 0.032) (Figure 3B). These results demonstrate that *CYP6M7* over-transcription alone is sufficient to confer resistance to both type I and type II pyrethroids.

### Heterologous expression of *CYP6M7*, *CYP6P9a*and *CYP6P9b*in *Escherichia coli*

Recombinant *CYP6M7* protein was successfully expressed at 21°C and 150 rpm and harvested 48 h after induction with δ-ALA and IPTG. The co-expression of the *CYP6M7* enzyme in *E. coli* produced CO-difference spectrum typical of a good-quality functional enzyme expressed predominantly as P450 with low P420 content. Previous attempts to express the recombinant enzyme from the *CYP6P9a* allele from field populations after co-transformation with cytochrome P450 reductase (CPR) into *E. coli* JM109 cells had been unsuccessful [4]. Here, successful expression was achieved by allowing the starter culture to grow until the optical density at 595 nm reached 0.7-0.8 and by increasing the final concentrations of IPTG and δ-ALA used for induction from 1 M and 0.5 M, respectively, to 1.25 M and 0.75 M. The expression temperature was 24°C for the first 24 h after induction and was then lowered to 18°C at 24–48 h. Optimal expression of *CYP6P9a* was obtained 36–48 h post-induction, as indicated by a good-quality CO-difference spectrum. Details of the expression of *CYP6P9b* were previously reported [4].

**Figure 4:**
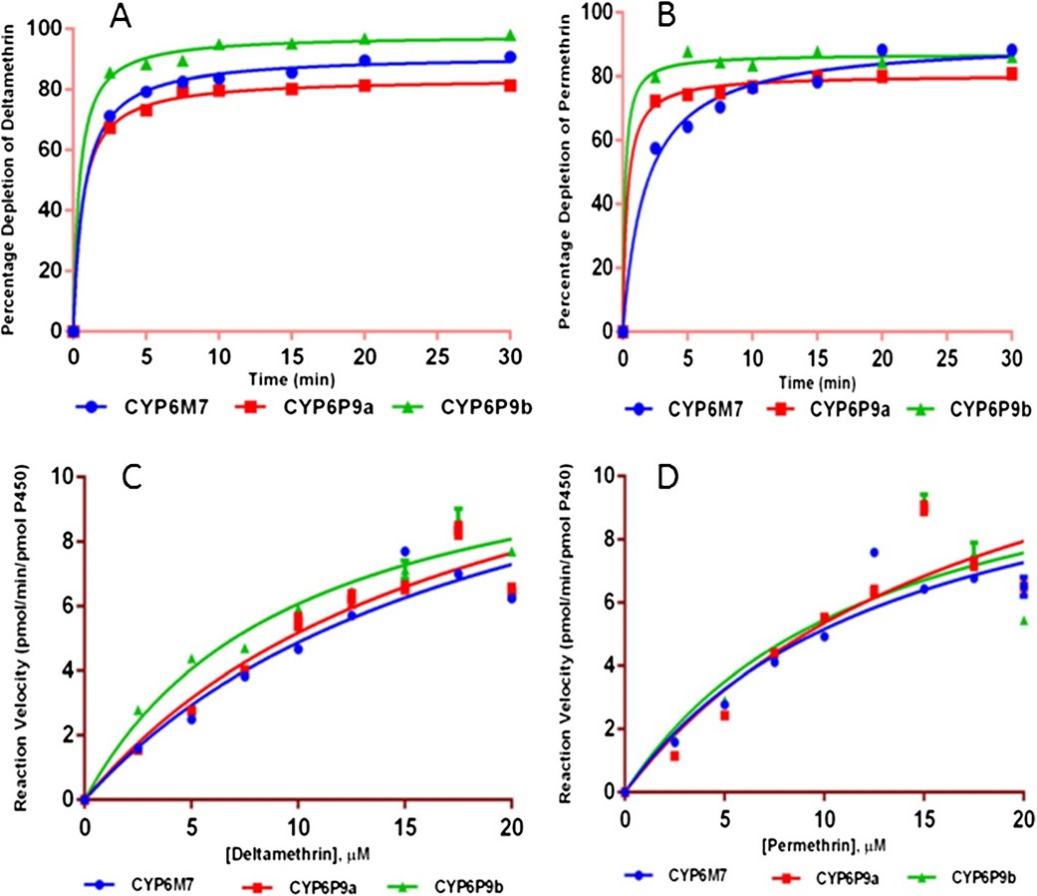
**Turnover and kinetic profiles of** ***CYP6P9a, CYP6P9b*** **and** ***CYP6M7*** **with type I and type II pyrethroids.** The turnover (time course) of the three enzymes with deltamethrin **(A)** and permethrin **(B)** is shown; **(C)** is the Michaelis-Menten plot of *CYP6P9a*, *CYP69b* and *CYP6M7* with deltamethrin, and **(D)** is the plot with permethrin. The data are presented as the mean ± S.D. of three replicates.

**Metabolism assays with*CYP6M7***: To confirm that *CYP6M7* can metabolize pyrethroid insecticides, the recombinant enzyme produced from this gene was assessed in *in vitro* metabolism assays. The metabolism assays demonstrated that recombinant *CYP6M7* metabolized both type I and type II pyrethroid insecticides (Figure 3C). Indeed, for the type I pyrethroids, significant substrate depletion rates of 68.28% ± 0.16 (P < 0.001) and 62.19% ± 0.6 (P < 0.001) were observed for permethrin and bifenthrin (as determined by the disappearance of substrate (20 μM) after 90 min), respectively, compared to the controls (with no NADPH). *CYP6M7* could also deplete the same concentration of the type II pyrethroids deltamethrin and lambda-cyhalothrin at rates of 53.60% ± 3.5 (P < 0.001) and 48.24% ± 1.97 (P < 0.001), respectively (Figure 4A). Similarly, *CYP6M7* can also metabolize the ether pyrethroid etofenprox with a significant depletion rate of 49.05% ±0.166 (P < 0.001).

**Metabolism assays with*CYP6P9a***: Similar to *CYP6P9b* [4], recombinant *CYP6P9a* also metabolized both type I and type II pyrethroids. A significant substrate depletion of 20 μM type I permethrin and bifenthrin was observed; 65.68% ± 1.5 (P < 0.001) and 68.48% ± 2.65 (P < 0.0001) of the substrate was deleted, respectively, by 90 minutes post-incubation. Similar significant depletion levels were also observed for the type II pyrethroids deltamethrin and lambda-cyhalothrin, with substrate disappearance rates of 68.4% ± 0.83 (P < 0.001) and 75.41% ± 0.50 (P < 0.001), respectively (Figure 4B). This enzyme also metabolized etofenprox with a depletion rate of 46.04% ± 0.26 (P < 0.0014).

### Turnover and kinetic profiles of *CYP6M7*, *CYP6P9a*and *CYP6P9b*

A comparative analysis of the turnover of the three enzymes during pyrethroid metabolism was performed. *CYP6P9a* produced a substrate turnover of 5.77 ± 1.48 min^-1^ and 5.91 ± 1.64 min^-1^ for permethrin and deltamethrin, respectively. *CYP6P9b* produced a turnover of 6.43 ± 1.40 min^-1^ and 7.041 ± 1.98 min^-1^ with permethrin and deltamethrin, respectively. *CYP6M7* followed the same pattern, with turnovers of 5.71 ± 1.52 min^-1^ for permethrin and 6.25 ± 1.67 min^-1^ for deltamethrin. These turnovers are within similar ranges, indicating that these three different enzymes have similar *in vitro* metabolic profiles with type I and type II pyrethroids.

Steady-state kinetic parameters for *CYP6M7*, *CYP6P9a* and *CYP6P9b* are given in Table 2 and Figure 4C and D. The K_M_ values obtained were within normal ranges associated with P450 metabolism (1–50 μM) in insects [28, 30, 39, 41] and in rats and mammals [42] but lower than those observed for *CYP6AA3* and *CYP6P7* in *An. minimus* with deltamethrin and permethrin [39].

**Table 2:** **Kinetic parameters for permethrin and deltamethrin metabolism by** ***CYP6M7, CYP6P9a*** **and** ***CYP6P9b***

Genes	Catalytic constant (Kcat) (min ^-1^)		K _M_(μM)		Catalytic efficiency (Kcat/Km) (min ^-1^ μM ^-1^)	
	Permethrin	Deltamethrin	Permethrin	Deltamethrin	Permethrin	Deltamethrin
**CYP6P9a**	8.11 ± 1.24	7.469 ± 1.25	18.77 ± 13.76	18.21 ± 8.904	0.432 ± 0.09	0.410 ± 0.141
**CYP6P9b**	8.37 ± 1.13	7.90 ± 1.10	12.68 ± 10.08	9.9 ± 3.653	0.660 ± 0.112	0.798 ± 0.303
**CYP6M7**	6.60 ± 0.24	7.035 ± 0.04	13.81 ± 7.075	19.64 ± 10.69	0.478 ± 0.034	0.358 ± 0.004

For *CYP6P9a*, the apparent K_M_ and rate constant (Kcat) were within the same range for deltamethrin and permethrin, suggesting comparable affinity and metabolic profiles for type I and type II pyrethroids. *CYP6M7* exhibited a lower K_M_ (higher affinity) for permethrin than deltamethrin, resulting in a higher catalytic efficiency (Kcat/K_M_) with permethrin. *CYP6P9b* had the highest affinity (lower K_M_) and highest Kcat for both pyrethroids compared with *CYP6P9a* and *CYP6M7*, which is reflected in its higher catalytic efficiency for deltamethrin and permethrin. This enzyme therefore metabolizes pyrethroids, particularly deltamethrin, with higher efficiency than both *CYP6P9a* and *CYP6M7* and other previously characterized P450s [28, 30, 39, 41]. Overall, for all three enzymes, the reactions appeared to follow Michaelis-Menten kinetics, with Kcat values within the range recorded in the literature for the activities of some insect P450s with pyrethroids [28, 30, 39, 41].

### Genetic variability of candidate resistance genes in relation to pyrethroid resistance

**Polymorphism analysis of*CYP6M7***: Analysis of the genetic variability of *CYP6M7* full-length cDNA (1503 bp) for five clones each from Mozambique, Malawi, Zambia and the susceptible FANG strain revealed a high polymorphism of this gene, with an average of 45 polymorphic sites observed for each sample and 22 amino acid changes observed in total. In contrast to *CYP6P9a* and *CYP6P9b* [4], no reduced variation in *CYP6M7* was observed in the resistant field samples compared to the susceptible strain FANG. This is reflected by the absence of specific clades based on resistance phenotype in the maximum likelihood phylogenetic tree (Additional file 2: Figure S2), in contrast to *CYP6P9a* and *CYP6P9b* [4].

**Figure 5:**
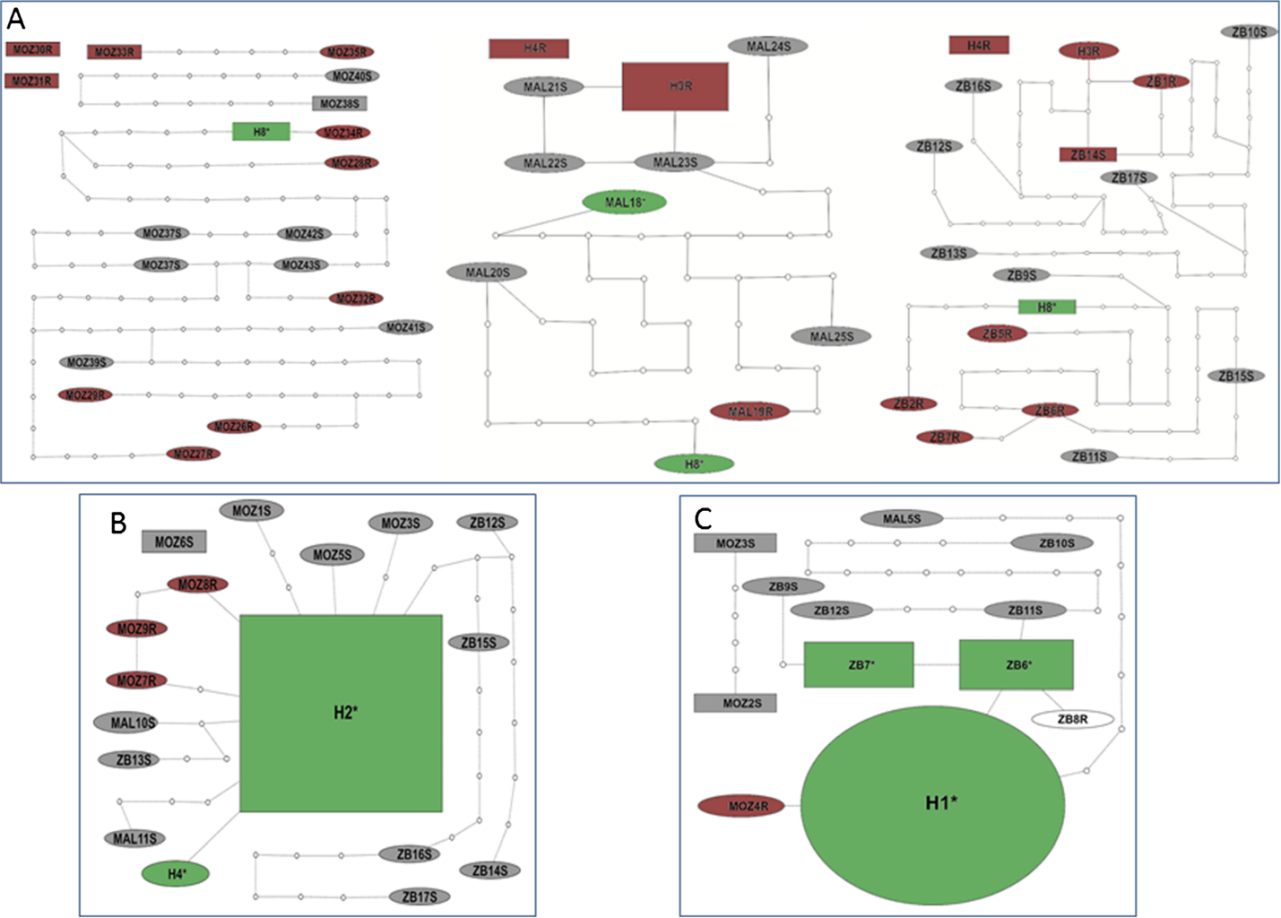
**Comparative analysis of haplotype diversity.** The haplotype diversities of *CYP6M7* **(A)** *CYP6P9a* **(B)** and CYP6P9b **(C)** were compared using a 95% parsimony network based only on coding regions when combining the susceptible (S) and resistant (R) mosquitoes from each country. For *CYP6M7,* networks are presented by country due to the large size of the combined network. These networks indicate the exceptional diversity of *CYP6M7* with high polymorphisms whereas *CYP6P9a* and *CYP6P9b* both exhibit reduced diversity, with the presence of a highly predominant haplotype associated with resistance (directional selection). Haplotypes are represented as an oval or a rectangle scaled to reflect their frequencies. The lines connecting haplotypes and each node represent a single mutation event. Gray shapes represent haplotypes unique in susceptible mosquitoes; green shapes represent haplotypes predominantly found in resistant mosquitoes but also in some dead mosquitoes; red shapes represent haplotypes unique to resistant mosquitoes. Some haplotypes with >20 mutation differences from others could not be linked to the major network.

**Comparative analysis of*CYP6M7*polymorphism in resistant and susceptible mosquitoes**: An analysis of sequence polymorphisms in a 2,148 bp genomic fragment spanning the full *CYP6M7* gene (2 exons and 1 intron) and a portion of the 5’ upstream region in five permethrin-resistant and five susceptible mosquitoes from each of the three countries revealed that *CYP6M7* is highly polymorphic. This high diversity of *CYP6M7* is reflected in the elevated number of substitution sites (226) and high haplotype diversity (51 haplotypes) detected across the three countries (Additional file 1: Table S4). *CYP6M7* polymorphism, based on the number of substitutions (S), the nucleotide diversity (π), haplotype number and haplotype diversity (hd), is significantly higher across the three countries than that of *CYP6P9a* and *CYP6P9b* (t-test, P < 0.05) (Additional file 2: Figure S3A, S3B and S3C). The higher polymorphism of *CYP6M7* is further supported by a high number of singleton haplotypes in *CYP6M7* (38 of 43 haplotypes for the coding region), with the most predominant haplotype in the three countries found only at a frequency of 15.5% (Additional file 2: Figure S3A), much lower than the equivalent frequencies of 63% for *CYP6P9a* (Additional file 2: Figure S3B) and 62% for *CYP6P9b* (Additional file 2: Figure S3C). A similar pattern was observed when the protein variants of these three genes were compared (Additional file 2: Figure S4A, S4B and S4C). In addition, the haplotype network of *CYP6M7* indicates that, in each of the three countries (Figure 5A) and in the total sample, *CYP6M7* haplotypes are more diverse than those of *CYP6P9a* (Figure 5B) and *CYP6P9b* (Figure 5C), as evidenced by an increase in the number of mutational steps (>50) separating haplotypes.

In contrast to *CYP6P9a* and *CYP6P9b*, no apparent correlation was observed between the genetic variability of *CYP6M7* and the resistance phenotype. Indeed, in the maximum likelihood phylogenetic tree of the haplotypes in each country, the haplotypes were not clustering according to their resistance phenotype (Additional file 2: Figure S5A), in contrast to the clustering observed for both *CYP6P9a* (Additional file 2: Figure S5B) and *CYP6P9b* (Additional file 2: Figure S5C). This lack of correlation between *CYP6M7* polymorphism and permethrin resistance is further supported by the similar levels of polymorphisms in both resistant and susceptible mosquitoes in the 3 countries, as measured by the number of polymorphic sites or by haplotypic (hd) and genetic (π) diversities (Additional file 1: Table S4 and Additional file 2: Figure S6A), which also differs from the findings for *CYP6P9a* (Additional file 2: Figure S6B) and *CYP6P9b* (Additional file 2: Figure S6C). However, sliding window analysis of the genetic diversity indicates a significant difference in polymorphism levels between susceptible and resistant samples for the 696 bp non-coding region (paired t-test, P < 0.01), with higher diversity among the total susceptible sample across the three countries compared to the resistant sample (Additional file 2: Figure S6D). Sliding window analysis for both *CYP6P9a* and *CYP6P9b* consistently revealed a higher diversity for susceptible mosquitoes across the entire gene (Additional file 2: Figure S6B and S6C).

Sliding window analysis of the genetic diversity of *CYP6M7* revealed that, as expected, this polymorphism is higher in the 5’ upstream non-coding region than the coding region for both the susceptible and resistant samples. Analysis of the coding region to detect polymorphism patterns associated with potential key functional regions of the gene detected three regions that exhibited higher diversity than the rest of the gene at positions 250–450 bp, 900–1100 bp and 1200–1350 bp (Additional file 2: Figure S6D, Additional file 1: Table S4).

***CYP6M7*selection test**: Patterns of selection acting on the coding region of *CYP6M7* were analyzed using the codon-based Z test of selection, which is based on the dN/dS ratio, as implemented in MEGA 5.2. A significant excess of synonymous substitutions per site (dS) over non-synonymous substitutions (dN) was consistently observed for both the susceptible and resistant samples from all countries for the analysis averaging over all sequence pairs within each group of samples (Additional file 1: Table S5). The codon-based test performed between sequence pairs also revealed that most Z values (dS-dN) were positive, irrespective of the resistance phenotype of the samples. A very high proportion of Z values (dS-dN) (64 to 98.4%) were associated with significant *P* values, supporting a significant excess of synonymous substitutions per site over non-synonymous substitutions (Additional file 1: Table S5).

**Polymorphisms of*CYP6P9a*and*CYP6P9b*in the Zambian sample**: Analysis of the polymorphism patterns of *CYP6P9a* and *CYP6P9b* in the Zambian sample revealed the same reduction of genetic diversity (number of substitutions, genetic diversity (π) and haplotype number) in resistant mosquitoes that was previously observed in the Malawian and Mozambican samples [4] (Additional file 1: Table S6 and S7). However, both genes were more variable in the Zambia sample than in the Mozambican and Malawian samples with notably more mutational steps between haplotypes. Indeed, only 1 haplotype of *CYP6P9a* was more than 4 mutational steps from any other in the Malawian and Mozambican samples, whereas in the Zambia sample, all haplotypes were between 5 to 18 mutational steps apart (Additional file 2: Figure S7A, S7B and S7C). In addition, the frequencies of the predominant haplotypes of both genes were lower in Zambia. This difference is more evident for the *CYP6P9b*, for which the predominant haplotype (H1) found in the Mozambican (84.5%; Additional file 2: Figure S7D) and Malawian (95%; Additional file 2: Figure S7E) samples is not present in the Zambian sample, which, by contrast, featured two predominant haplotypes, ZB6 (40%) and ZB7 (35%) (Additional file 2: Figure S7F). These haplotypes were separated by 1 and 2 mutations, respectively, from haplotype H1 (Figure 5C). Furthermore, analysis of the maximum likelihood tree of *CYP6P9a* and *CYP6P9b* haplotypes detected a cluster of haplotypes only specific to Zambia (Additional file 2: Figure S5B and S5C). The differences observed in Zambia for both genes resulted in a higher and significant genetic differentiation between Zambia and the two other countries based on *K*_*ST*_ estimates whereas no significant differentiation is observed between Malawi and Mozambique (Additional file 1: Table S8). In contrast, for *CYP6M7* all populations were significantly differentiated from each other. These patterns are confirmed by the phylogenetic trees based on genetic distances (Additional file 2: Figure S5D, S5E and S5F).

## Discussion

Insecticide resistance among *Anopheles* mosquitoes is spreading throughout Africa, threatening the success of malaria control methods. In this study, after characterizing the insecticide resistance profile of an *An. funestus* population from Zambia, we elucidated the molecular and genetic basis of the resistance mechanisms driving the expansion of the pyrethroid resistance front across Southern Africa.

### Expansion of the resistance front in *An. funestus*is likely due to multiple independent selection events

The rapid spread of insecticide resistance from a single localized origin to a large geographical region due to extensive gene flow between vector populations is of great concern to control programs [2]. Determining whether one or more resistance profiles are present can facilitate the design and implementation of successful vector control strategies. The comparative analysis of the susceptibility profile of the Zambian population with those of the Mozambican and Malawian populations suggests that the resistance observed in Southern Africa might not have developed from a single origin and spread over the region as previously suggested based on the similarity between Mozambican and Malawian populations [4, 8]. Local selection factors probably play an important role in the expansion of this resistance as highlighted by the significant differences in the resistance profiles of the Zambian population compared with the Malawian and Mozambican populations. The DDT resistance observed in the Zambian population contrasts with the full susceptibility to this insecticide in the Mozambican population and the very moderate resistance in the Malawian population, suggesting that local selection factors in Zambia (from agriculture or from public health control interventions such as IRS) could be responsible for this DDT resistance. The higher carbamate resistance of the Zambian sample compared to the Malawian and Mozambican samples further supports this observation. In *An. gambiae*, local selection factors have played a similarly important role in the development of resistance, such as in the highly selected agricultural settings in Burkina Faso [26, 43] and in Benin [12]. However, the rapid spread of the 1014 F *kdr* mutation in *An. gambiae* [44] is a reminder that, with time, gene flow between populations will also contribute to such resistance expansion.

The variation in the susceptibility profile and resistance genes observed in this study highlights the need to characterize local populations before designing and implementing control strategies because information from a single country is insufficient to guide decision-making in a neighboring country.

### Expansion of pyrethroid resistance is driven by new resistance genes: a challenge for the management of metabolic resistance

This study has revealed that beside the previously confirmed P450s *CYP6P9a* and *CYP6P9b* resistance genes, the *CYP6M7* gene is likely also playing an important role in the pyrethroid resistance in southern Africa as shown by a consistent over-expression from microarray. The fact that similar consistent over-expression for *CYP6M7* was observed from the qRT-PCR results using three different primer pairs further validates the up-regulation of this gene in relation to pyrethroid resistance. Interestingly, probes from *CYP6M3*, the ortholog of *CYP6M7* in *An. gambiae*, were found to be significantly over-expressed in the pyrethroid resistant laboratory *An. funestus* FUMOZ strain compared to the laboratory susceptible FANG strain during a microarray analysis using the *An. gambiae* "detox chip" [45]. *CYP6M7* was also previously mapped within the *rp2* pyrethroid resistance QTL in a crossing between FUMOZ and FANG strains [21]. Altogether, the consistent over-expression of *CYP6M7* and the demonstration that this gene can metabolize pyrethroids both *in vitro* and *in vivo*, support the view that *CYP6M7* is significantly contributing to pyrethroid resistance in southern Africa. The two to seven-fold reduced expression of both *CYP6P9a* and *CYP6P9b* in Zambia from microarray in contrast to Mozambique and Malawi, could even signal a possible shift in underlying molecular basis of pyrethroid resistance across the region with *CYP6M7* possibly playing a more important in such locations. However, it will be useful in future studies to further validate this potential shift in gene expression of the main resistance genes using other methods such as RNAseq which contrary to microarray and qRT-PCR is not affected by the sequence polymorphism of targeted genes. The reasons for the potential shift in the expression levels of the three genes remain unknown but could be due to the nature of the selection that gave rise to the resistance. This shift in gene expression further highlights the genetic plasticity of natural populations of malaria vectors and their ability to adapt to various selection pressures. Similar geographical variations in expression levels between detoxification genes have been observed in other mosquito vectors, such as *An. gambiae* [38] and *Aedes aegypti* [46]. Furthermore, it is not uncommon for different P450 genes to be associated with a same resistance profile such as the case for the *CYP6A1* and *CYP6D1* in the house fly *Musca domestica*, or for *CYP6A2*, *CYP6A8* and *CYP6G1* in the fruit fly *D. melanogaster* [47]. Similarly in the red flour beetle *Tribolium castaneum* several P450s including *CYP6BQ8*, *CYP6BQ9* and *CYP6BQ10* or *CYP436B1 and CYP436B2*, are shown to confer deltamethrin resistance [40, 48].

Functional characterization of the three cytochrome P450 genes clearly demonstrated that they all confer to *An. funestus* the ability to withstand both types I and II pyrethroids. It will be a concern for control programs if the three genes were equally highly up-regulated in a single population, as this may significantly increase the overall pyrethroid resistance level of such mosquito populations.

However, kinetic analysis of the three recombinant enzymes suggests that *CYP6P9b* may metabolize pyrethroids more efficiently than the other two enzymes. This result will need to be further confirmed since kinetic profiles obtained through *in vitro* experiments are unlikely to reflect 100% their *in vivo* patterns stemming from potential variations in the *in vivo* intrinsic clearance of the insecticide in the mosquito. Variations in insecticide metabolism among resistance genes are not uncommon and were previously observed in *D. melanogaster* when seven P450 genes were compared for their ability to confer resistance using transgenic expression [49]. However, more work is needed to fully establish the metabolic efficiency of these enzymes against all pyrethroids. Another area of investigation will be to establish their patterns of cross-resistance to other insecticide classes. Such P450 cross-resistance to different insecticide classes was recently reported in *An. gambiae* for the *CYP6M2* gene [13] or for the *CYP6G1* in *D. melanogaster* which has been showed to metabolize a wide range of insecticides [50]. Similarly, it will be interesting to determine if the increased *CYP6M7* expression in the Zambian population also plays a role in the DDT resistance or bendiocarb resistance observed in this country.

The change in the resistance profile observed in Zambia was also reflected in the transcription profile of this mosquito population with the over-expression of more GSTs and carboxylesterases in this country. The low over-expression of the *GSTe2* gene, along with the absence of the *GSTe2* L119F mutation commonly associated with DDT resistance in West Africa, suggest that the mechanisms of DDT resistance in Zambia are different than those observed in West and Central Africa [51]. The seven-fold over-expression of the *GSTD3* in Zambia than in the DDT-susceptible populations of Mozambique and Malawi could suggest that the up-regulation of this gene could be associated with the DDT resistance in Zambia. However, further work is needed to establish the exact contribution of this gene and others with the resistance observed against pyrethroids or DDT and bendiocarb in this study. The contribution of several genes to metabolic resistance to insecticides is a common trait in mosquitoes and is also observed in other vectors such as *An. gambiae*, in which the P450 gene, *CYP6P3*, combines with *CYP6M2* to confer pyrethroid resistance in field populations [12–14]. Similar phenomena have been observed in *Ae. aegypti*, in which a set of CYP9 P450s play a major role in resistance [52, 53], and even in *Drosophila*, in which many genes combine to protect against various spectrums of insecticides [49]. The involvement of a multiplicity of genes in metabolic resistance is a further challenge to the ultimate aim of detecting resistance markers that could be used to design suitable DNA-based diagnostic tools to easily detect such resistance in the field.

### Contrasting selection patterns shape the diversity of *CYP6M7*and the duplicated *CYP6P9a*and *CYP6P9b*

*CYP6M7* is highly polymorphic, with 1 SNP every 15 bp in the coding regions and 1 SNP every 9 bp when non-coding regions are included. This level of polymorphism far exceeds that previously reported for any P450 gene in *An. funestus* [21, 54] or *An. gambiae* [55]. Analysis of the genetic diversity of *CYP6M3*, the *CYP6M7* ortholog in *An. gambiae*, revealed a far lower polymorphism level, with only 1 SNP every 66 bp [55]. The high polymorphism of *CYP6M7* is similar to that reported in *An. gambiae* for the *APL1* gene which is associated with immune responses against *Plasmodium falciparum* [56]. It is unlikely that the high polymorphism of *CYP6M7* is associated with gene duplication, as good and clean sequencing results were consistently obtained from PCR products of same size after amplification using primers located in the 5’ and 3’UTR. In addition, a full sequencing of the 113 kb of this genomic region spanning *rp2* pyrethroid QTL did not detect any gene duplication of *CYP6M7* [21].

No particular *CYP6M7* allele seems to be most associated with pyrethroid resistance, in contrast to the identification of predominant resistance alleles of *CYP6P9a* and *CYP6P9b* [4] suggesting that no selective sweep is acting upon *CYP6M7*. This suggests that the major genetic factor conferring resistance through *CYP6M7* could be located in a trans regulatory region rather than in the coding sequence, but this regulator remains to be found. By contrast, the considerable differences observed in the amino acid sequences of *CYP6P9a* and *CYP6P9b* between resistant and susceptible mosquitoes suggests that allelic variation at these two genes impact their ability to confer pyrethroid resistance. Further work on the regulation pathway of *CYP6M7* will help to shed more light into why it has not experienced the same selective sweep as *CYP6P9a* and *CYP6P9b*.

The high polymorphism of *CYP6M7* was nevertheless associated with a significant predominance of synonymous substitutions over non-synonymous substitutions suggesting that *CYP6M7* amino acid sequence was under some selective constraints. However, the high polymorphism level observed for *CYP6M7* and the lack of predominant haplotype suggests that *CYP6M7* is overall probably evolving neutrally. This is in contrast to both *CYP6P9a* and *CYP6P9b* were a signature of directional selection is observed with the selection of a predominant haplotype and a reduced polymorphism in resistance mosquitoes in all three countries. Beside a possible trans-regulation of *CYP6M7*, the absence of a directional selection on *CYP6M7* could also stem from a more generalist functional role of this gene, particularly if it could protect the mosquito against a broad range of xenobiotics or against plant toxins (allelochemicals) or even if it were involved in functions other than detoxification. This could explain why a single allele has not been favored. By contrast, the directional selection acting on both *CYP6P9a* and *CYP6P9b* with a selection of favorable alleles could be due to a more specialist role of these genes against mainly pyrethroid insecticides. Similarly, structural differences associated with sequence polymorphisms have been observed between the generalist cytochrome P450 *CYP6B8* (which is able to metabolize a variety of allelochemicals and insecticides) in the polyphagous noctuid *Helicoverpa zea* and the specialist *CYP6B1* in the black swallowtail butterfly *Papilio polyxenes*, a specialist restricted to furanocoumarin-containing plants [57]. Further evaluation of the substrate spectrum metabolism of *CYP6M7*, *CYP6P9a* and *CYP6P9b* is needed to confirm this hypothesis.

The higher genetic diversity of *CYP6P9a* and *CYP6P9b* in the Zambian sample compared to the Malawian and Mozambican samples correlates with the lower expression of both genes in the Zambian sample. This supports the conclusion that both genes could be under less selective pressure in Zambia. This variation in the diversity of both genes in relation to their role in pyrethroid resistance is similar to the differences in the genetic variability of the glutathione S-transferase *GSTe2* in *An. funestus* with DDT resistance across Africa [51]. Very low genetic variability is observed in high DDT resistance regions, whereas higher variability is observed in more susceptible regions [51]. A similar distribution of variation in the *CYP6G1* P450 gene has been associated with DDT resistance in *Drosophila simulans*. This gene has significantly lower diversity in DDT-resistant populations from California and higher polymorphism in DDT-susceptible populations from Zimbabwe [58].

## Conclusion

Taken together, the results from this study have helped to shed more light into the molecular complexities of the resistance mechanisms underlying pyrethroid resistance in southern African populations of *An. funestus*. Indeed, this study has revealed that beside the previously known resistance genes *CYP6P9a* and *CYP6P9b*, the *CYP6M7* P450 gene is also playing a key role in the pyrethroid resistance observed in the region and that its role may even become more important in the northern range of the resistance front such as in Zambia. Therefore, despite the similarities observed, the underlying molecular basis of pyrethroid resistance is probably not uniform across its distribution range in Southern Africa. The northern range in Zambia exhibits significant differences from Malawi and Mozambique in term of the profile and molecular basis of resistance. It is therefore likely that beyond Mozambique and Malawi, pyrethroid resistance in southern Africa has multiple origins under different evolutionary forces, which may necessitate the design of different resistance management strategies to mitigate the impact of this resistance.

## Electronic supplementary material

Additional file 1: Table S1: List of primers used in this study. **Table S2.** Top 50 probes the most commonly up-regulated in R-S in Mozambique, Malawi and Zambia. **Table S3.** Top 20 probes the most commonly down-regulated in R-S in Mozambique, Malawi and Zambia. **Table S4.** Summary statistics for polymorphism of *CYP6M7* between susceptible and resistant mosquitoes in Zambia, Malawi and Mozambique. **Table S5.** Codon-based Test of Selection for *CYP6M7* for analysis averaging over all sequence pairs within each group. **Table S6.** Summary statistics for polymorphism of *CYP6P9a* between susceptible and resistant mosquitoes in Zambia, Malawi and Mozambique. **Table S7.** Summary statistics for polymorphism of *CYP6P9b* between susceptible and resistant mosquitoes in Zambia, Malawi and Mozambique. **Table S8.** Genetic differentiation using *KST* for *CYP6P9a*, *CYP6P9b* and *CYP6M7*. (PDF 315 KB)

Additional file 2: Figure S1: Gene expression analysis. **Figure S2.** Maximum likelihood (ML) tree of full-length *CYP6M7* cDNA haplotypes from various regions of Africa. **Figure S3.** Analysis of polymorphisms of *CYP6M7*, *CYP6P9a* and *CYP6P9b*. **Figure S4.** Protein variants among resistant and susceptible mosquitoes from all three countries. **Figure S5.** Phylogenetic analysis of *CYP6M7*. **Figure S6.** Impact of permethrin resistance on genetic diversity. **Figure S7.** Comparative analysis of the haplotypes of the three genes based only on coding regions among susceptible and resistant mosquitoes from the three countries using a 95% parsimony network. (PDF 638 KB)

## References

[1] WHO (2011). Malaria Report 2011.

[2] WHO (2012). Global Plan for Insecticide Resistance Management (GPIRM).

[3] Okoye PN, Brooke BD, Koekemoer LL, Hunt RH, Coetzee M (2008). Characterisation of DDT, pyrethroid and carbamate resistance in Anopheles funestus from Obuasi, Ghana. Trans R Soc Trop Med Hyg.

[4] Riveron JM, Irving H, Ndula M, Barnes KG, Ibrahim SS, Paine MJ, Wondji CS (2013). Directionally selected cytochrome P450 alleles are driving the spread of pyrethroid resistance in the major malaria vector Anopheles funestus. Proc Natl Acad Sci U S A.

[5] Wondji CS, Coleman M, Kleinschmidt I, Mzilahowa T, Irving H, Ndula M, Rehman A, Morgan J, Barnes KG, Hemingway J (2012). Impact of pyrethroid resistance on operational malaria control in Malawi. Proc Natl Acad Sci U S A.

[6] Amenya DA, Naguran R, Lo TC, Ranson H, Spillings BL, Wood OR, Brooke BD, Coetzee M, Koekemoer LL (2008). Over expression of a cytochrome P450 (CYP6P9) in a major African malaria vector, Anopheles Funestus, resistant to pyrethroids. Insect Mol Biol.

[7] Chanda E, Hemingway J, Kleinschmidt I, Rehman AM, Ramdeen V, Phiri FN, Coetzer S, Mthembu D, Shinondo CJ, Chizema-Kawesha E, Kamuliwo M, Mukonka V, Baboo KS, Coleman M (2011). Insecticide resistance and the future of malaria control in zambia. PLoS One.

[8] Hunt R, Edwardes M, Coetzee M (2010). Pyrethroid resistance in southern African Anopheles funestus extends to Likoma Island in Lake Malawi. Parasit Vectors.

[9] Schlenke TA, Begun DJ (2004). Strong selective sweep associated with a transposon insertion in Drosophila simulans. Proc Natl Acad Sci U S A.

[10] Martinez-Torres D, Chandre F, Williamson MS, Darriet F, Berge JB, Devonshire AL, Guillet P, Pasteur N, Pauron D (1998). Molecular characterization of pyrethroid knockdown resistance (kdr) in the major malaria vector Anopheles gambiae s.s. Insect Mol Biol.

[11] Ranson H, Jensen B, Vulule JM, Wang X, Hemingway J, Collins FH (2000). Identification of a point mutation in the voltage-gated sodium channel gene of Kenyan Anopheles gambiae associated with resistance to DDT and pyrethroids. Insect Mol Biol.

[12] Djouaka RF, Bakare AA, Coulibaly ON, Akogbeto MC, Ranson H, Hemingway J, Strode C (2008). Expression of the cytochrome P450s, CYP6P3 and CYP6M2 are significantly elevated in multiple pyrethroid resistant populations of Anopheles gambiae s.s. from Southern Benin and Nigeria. BMC Genomics.

[13] Mitchell SN, Stevenson BJ, Muller P, Wilding CS, Egyir-Yawson A, Field SG, Hemingway J, Paine MJ, Ranson H, Donnelly MJ (2012). Identification and validation of a gene causing cross-resistance between insecticide classes in Anopheles gambiae from Ghana. Proc Natl Acad Sci U S A.

[14] Muller P, Warr E, Stevenson BJ, Pignatelli PM, Morgan JC, Steven A, Yawson AE, Mitchell SN, Ranson H, Hemingway J, Paine MJ, Donnelly MJ (2008). Field-caught permethrin-resistant Anopheles gambiae overexpress CYP6P3, a P450 that metabolises pyrethroids. PLoS Genet.

[15] Gregory R, Darby AC, Irving H, Coulibaly MB, Hughes M, Koekemoer LL, Coetzee M, Ranson H, Hemingway J, Hall N, Wondji CS (2011). A De novo expression profiling of anopheles funestus, malaria vector in Africa, using 454 pyrosequencing. PLoS One.

[16] Crawford JE, Guelbeogo WM, Sanou A, Traore A, Vernick KD, Sagnon N, Lazzaro BP (2010). De novo transcriptome sequencing in Anopheles funestus using Illumina RNA-seq technology. PLoS One.

[17] Cuamba N, Morgan JC, Irving H, Steven A, Wondji CS (2010). High level of pyrethroid resistance in an Anopheles funestus population of the Chokwe District in Mozambique. PLoS One.

[18] Gillies MT, Coetzee M (1987). A supplement to the Anophelinae of Africa south of the Sahara (Afrotropical region), Volume 55.

[19] Koekemoer LL, Kamau L, Hunt RH, Coetzee M (2002). A cocktail polymerase chain reaction assay to identify members of the Anopheles funestus (Diptera: Culicidae) group. Am J Trop Med Hyg.

[20] WHO (1998). Test procedures for insecticide resistance montoring in malaria vectors, bio-efficacy and persistence of insecticides on treated surfaces.

[21] Irving H, Riveron JM, Ibrahim SS, Lobo NF, Wondji CS (2012). Positional cloning of rp2 QTL associates the P450 genes CYP6Z1, CYP6Z3 and CYP6M7 with pyrethroid resistance in the malaria vector Anopheles funestus. Heredity (Edinb).

[22] Wondji CS, Irving H, Morgan J, Lobo NF, Collins FH, Hunt RH, Coetzee M, Hemingway J, Ranson H (2009). Two duplicated P450 genes are associated with pyrethroid resistance in Anopheles funestus, a major malaria vector. Genome Res.

[23] David JP, Strode C, Vontas J, Nikou D, Vaughan A, Pignatelli PM, Louis C, Hemingway J, Ranson H (2005). The Anopheles gambiae detoxification chip: a highly specific microarray to study metabolic-based insecticide resistance in malaria vectors. Proc Natl Acad Sci U S A.

[24] Conesa A, Gotz S, Garcia-Gomez JM, Terol J, Talon M, Robles M (2005). Blast2GO: a universal tool for annotation, visualization and analysis in functional genomics research. Bioinformatics.

[25] Gotz S, Garcia-Gomez JM, Terol J, Williams TD, Nagaraj SH, Nueda MJ, Robles M, Talon M, Dopazo J, Conesa A (2008). High-throughput functional annotation and data mining with the Blast2GO suite. Nucleic Acids Res.

[26] Kwiatkowska RM, Platt N, Poupardin R, Irving H, Dabire RK, Mitchell S, Jones CM, Diabate A, Ranson H, Wondji CS (2013). Dissecting the mechanisms responsible for the multiple insecticide resistance phenotype in Anopheles gambiae s.s., M form, from Vallee du Kou, Burkina Faso. Gene.

[27] Schmittgen TD, Livak KJ (2008). Analyzing real-time PCR data by the comparative C-T method. Nat Protoc.

[28] McLaughlin LA, Niazi U, Bibby J, David JP, Vontas J, Hemingway J, Ranson H, Sutcliffe MJ, Paine MJ (2008). Characterization of inhibitors and substrates of Anopheles gambiae CYP6Z2. Insect Mol Biol.

[29] Pritchard MP, Glancey MJ, Blake JA, Gilham DE, Burchell B, Wolf CR, Friedberg T (1998). Functional co-expression of CYP2D6 and human NADPH-cytochrome P450 reductase in Escherichia coli. Pharmacogenetics.

[30] Stevenson BJ, Bibby J, Pignatelli P, Muangnoicharoen S, O'Neill PM, Lian LY, Muller P, Nikou D, Steven A, Hemingway J, Sutcliffe MJ, Paine MJ (2011). Cytochrome P450 6 M2 from the malaria vector Anopheles gambiae metabolizes pyrethroids: Sequential metabolism of deltamethrin revealed. Insect Biochem Mol Biol.

[31] Omura T, Sato R (1964). The carbon monoxide-binding pigment of liver microsomes. I Evidence for Its Hemoprotein Nature J Biol Chem.

[32] Strobel HW, Dignam JD (1978). Purification and properties of NADPH-cytochrome P-450 reductase. Methods Enzymol.

[33] Thompson JD, Higgins DG, Gibson TJ (1994). CLUSTAL W: improving the sensitivity of progressive multiple sequence alignment through sequence weighting, position-specific gap penalties and weight matrix choice. Nucleic Acids Res.

[34] Rozas J, Sanchez-DelBarrio JC, Messeguer X, Rozas R (2003). DnaSP, DNA polymorphism analyses by the coalescent and other methods. Bioinformatics.

[35] Tamura K, Dudley J, Nei M, Kumar S (2007). MEGA4: molecular Evolutionary Genetics Analysis (MEGA) software version 4.0. Mol Biol Evol.

[36] Clement M, Posada D, Crandall KA (2000). TCS: a computer program to estimate gene genealogies. Mol Ecol.

[37] Nei M, Gojobori T (1986). Simple methods for estimating the numbers of synonymous and nonsynonymous nucleotide substitutions. Mol Biol Evol.

[38] Fossog Tene B, Poupardin R, Costantini C, Awono-Ambene P, Wondji CS, Ranson H, Antonio-Nkondjio C (2013). Resistance to DDT in an urban setting: common mechanisms implicated in both M and S forms of Anopheles gambiae in the city of Yaounde Cameroon. PLoS One.

[39] Duangkaew P, Pethuan S, Kaewpa D, Boonsuepsakul S, Sarapusit S, Rongnoparut P (2011). Characterization of mosquito CYP6P7 and CYP6AA3: differences in substrate preference and kinetic properties. Arch Insect Biochem Physiol.

[40] Feyereisen R, Insect Molecular Biology and Biochemistry (2011). Insect CYP Genes and P450 enzymes. Gilbert LI.

[41] Stevenson BJ, Pignatelli P, Nikou D, Paine MJ (2012). Pinpointing P450s associated with pyrethroid metabolism in the dengue vector, Aedes aegypti: developing new tools to combat insecticide resistance. PLoS Negl Trop Dis.

[42] Scollon EJ, Starr JM, Godin SJ, DeVito MJ, Hughes MF (2009). In vitro metabolism of pyrethroid pesticides by rat and human hepatic microsomes and cytochrome p450 isoforms. Drug Metab Dispos.

[43] Dabiré KR, Diabaté A, Namountougou M, Djogbenou L, Wondji C, Chandre F, Simard F, Ouédraogo J-B, Martin T, Weill M, Baldet T, Parveen F (2012). Trends in Insecticide Resistance in Natural Populations of Malaria Vectors in Burkina Faso, West Africa: 10 Years’ Surveys. Pest Engineering, Volume Insecticides-Pest Engineering.

[44] Ranson H, N'Guessan R, Lines J, Moiroux N, Nkuni Z, Corbel V (2011). Pyrethroid resistance in African anopheline mosquitoes: what are the implications for malaria control?. Trends Parasitol.

[45] Christian RN, Strode C, Ranson H, Coetzer N, Coetzee M, Koekemoer LL (2011). Microarray analysis of a pyrethroid resistant African malaria vector, Anopheles funestus, from southern Africa. Pestic Biochem Physiol.

[46] Bariami V, Jones CM, Poupardin R, Vontas J, Ranson H (2012). Gene amplification, ABC transporters and cytochrome P450s: unraveling the molecular basis of pyrethroid resistance in the dengue vector. Aedes aegypti PLoS Negl Trop Dis.

[47] Li X, Schuler MA, Berenbaum MR (2007). Molecular mechanisms of metabolic resistance to synthetic and natural xenobiotics. Annu Rev Entomol.

[48] Zhu F, Parthasarathy R, Bai H, Woithe K, Kaussmann M, Nauen R, Harrison DA, Palli SR (2010). A brain-specific cytochrome P450 responsible for the majority of deltamethrin resistance in the QTC279 strain of Tribolium castaneum. Proc Natl Acad Sci U S A.

[49] Daborn PJ, Lumb C, Boey A, Wong W, Ffrench-Constant RH, Batterham P (2007). Evaluating the insecticide resistance potential of eight Drosophila melanogaster cytochrome P450 genes by transgenic over-expression. Insect Biochem Mol Biol.

[50] Ffrench-Constant RH (2013). The molecular genetics of insecticide resistance. Genetics.

[51] Riveron JM, Yunta C, Ibrahim SS, Djouaka R, Irving H, Menze BD, Ismail HM, Hemingway J, Ranson H, Albert A, Wondji CS (2014). A single mutation in the GSTe2 gene allows tracking of metabolically-based insecticide resistance in a major malaria vector. Genome Biol.

[52] Strode C, Wondji CS, David JP, Hawkes NJ, Lumjuan N, Nelson DR, Drane DR, Karunaratne SH, Hemingway J, Black WC, Ranson H (2008). Genomic analysis of detoxification genes in the mosquito Aedes aegypti. Insect Biochem Mol Biol.

[53] Marcombe S, Poupardin R, Darriet F, Reynaud S, Bonnet J, Strode C, Brengues C, Yebakima A, Ranson H, Corbel V, David JP (2009). Exploring the molecular basis of insecticide resistance in the dengue vector Aedes aegypti: a case study in Martinique Island (French West Indies). BMC Genomics.

[54] Wondji CS, Hemingway J, Ranson H (2007). Identification and analysis of Single Nucleotide Polymorphisms (SNPs) in the mosquito Anopheles funestus, malaria vector. BMC Genomics.

[55] Wilding CS, Weetman D, Steen K, Donnelly MJ (2009). High, clustered, nucleotide diversity in the genome of Anopheles gambiae revealed through pooled-template sequencing: implications for high-throughput genotyping protocols. BMC Genomics.

[56] Rottschaefer SM, Riehle MM, Coulibaly B, Sacko M, Niare O, Morlais I, Traore SF, Vernick KD, Lazzaro BP (2011). Exceptional diversity, maintenance of polymorphism, and recent directional selection on the APL1 malaria resistance genes of Anopheles gambiae. PLoS Biol.

[57] Li X, Baudry J, Berenbaum MR, Schuler MA (2004). Structural and functional divergence of insect CYP6B proteins: from specialist to generalist cytochrome P450. Proc Natl Acad Sci U S A.

