# Supplementary material for: The highly polymorphic CYP6M7 cytochrome P450 gene partners with the directionally selected CYP6P9a and CYP6P9b genes to expand the pyrethroid resistance front in the malaria vector Anopheles funestus in Africa
Source: BMC Genomics. 2014 Sep 27;15(1):817. doi: 10.1186/1471-2164-15-817 (PMC4192331; doi:10.1186/1471-2164-15-817)
Supplement: Supplementary file 1 — Additional file 1: Table S1: List of primers used in this study. Table S2. Top 50 probes the most commonly up-regulated in R-S in Mozambique, Malawi and Zambia. Table S3. Top 20 probes the most commonly down-regulated in R-S in Mozambique, Malawi and Zambia. Table S4. Summary statistics for polymorphism of CYP6M7 between susceptible and resistant mosquitoes in Zambia, Malawi and Mozambique. Table S5. Codon-based Test of Selection for CYP6M7 for analysis averaging over all sequence pairs within each group. Table S6. Summary statistics for polymorphism of CYP6P9a between susceptible and resistant mosquitoes in Zambia, Malawi and Mozambique. Table S7. Summary statistics for polymorphism of CYP6P9b between susceptible and resistant mosquitoes in Zambia, Malawi and Mozambique. Table S8. Genetic differentiation using KST for CYP6P9a, CYP6P9b and CYP6M7. (PDF 315 KB) [file 12864_2014_6509_MOESM1_ESM.pdf]

Table S1: List of primers used in this study

Gene	Forward primer	Reverse primer	Expected size (bp)
		**Primers used for qRT-PCR**	
**CYP6P9a**	CAGCGCGTACACCAGATTGTGTAA	TCACAATTTTTCCACCTTCAAGTAATTACCCGC	92
**CYP6P9b**	CAGCGCGTACACCAGATTGTGTAA	TTACACCTTTTCTACCTTCAAGTAATTACCCGC	97
**CYP6AA1**	CATCTGGCTGAATGGCACTA	TCAACAATGCCATCAAATCG	109
**CYP9J11**	CAAATTTAAAGAGTGCGCTAGG	GTAGATGGTGCCAAGGATGG	115
**CYP6Z1**	GGATTTCCGATGAGGATTGA	GCAGCGTACTTGATTTACGG	78
**CYP9K1**	AGGGCTTCTGGATACGGTTC	CGTACGGTTCGGTTTTGATT	103
**CYP6P4A**	AACTCGTATTCGACCCCAAA	CGTTTCCATGGAATTACATTTTCTG	146
**CYP6M4**	CACTATTCTCTCGCCGAAGG	CAAAGGATCCGCCATTCTAC	119
**CYP6N1**	GAAGCATTTCCGTTTTACGC	GGTGGCTTTATAGCTCGTT	138
**Arg-Lyase**	ATTCGTCGATGGGCATGTA	CACTTTTCACGGATCCTTTTG	120
**Combined_C3440**	GCCTTTTGGATCCTTGACCT	TAAAGCCCCAAAACAACAGG	150
**Ald Oxi**	GACTGGCAGACGATTGGATT	TGTAATCCAGCAACGGTGTC	134
**GSTe2**	GTTTGAAGCAGTTGCCATACTACGAGG	TCAAGCTTTAGCATTTTCCTCCTTTTTGGC	101
**CYP304B1**	GTTTCTGACGTTGGCAGCTT	CCGGTGCGGCTTTATCTC	150
**RSP7**	GTGTTCGGTTCCAAGGTGAT	TCCGAGTTCATTTCCAGCTC	98
**ACTIN**	TTAAACCCAAAAGCCAATCG	ACCGGATGCATACAGTGACA	111
**CYP6M7-3**	ACGACGGTACGCTAACGACT	TAACGCCAGCTCATACAACG	113
**CYP6M7-4**	CGTTGTATGAGCTGGCGTTA	GTGCATCTCCATGACAGCAT	116
**CYP6M7-6**	GGTTGGCCTACTTGCTAACG	AGCTTTTCCACCTTCAACCA	127
		**Primers used for functional characterisation**	
**CYP6P9afull**	ATGGAGCTCATTAACGTGGTGTTGGC	TCA CAA TTT TTC CAC CTT CAA GTA ATT ACC CGC	
**CYP6P9bfull**	ATGGAGCTCATTAACGTGGTGTTGGC	TTA CAC CTT TTC TAC CTT CAA GTA ATT ACC CGC	
**CYP6M7full**			
**CYP6P9a-pUAST**	AGATCTATGGAGCTCATTAACGTGGTG	TCTAGATCACAATTTTTCCACCTTCAAGTAA	
**CYP6P9b-pUAST**	AGATCTATGGAGCTCATTAACGTGGTG<br>TT	TCTAGACTACAAAAACCCCTTCCGCT	

**CYP6M7-pUAST**	TTGAATTCATGGAGCCGCTAGACATTTTGA	CCTCTAGATCATGTGCTCAGCTTTTCCACC	
**OMPA+2 FORWARD**	GGAATTCCATATGAAAAAGACAGCTATCGCG		
**OMPA+2 CYP6P9a/bF**	CAACACCACGTTAATGAGCTCCATCGG AGCGGCCTGCGCTACGGTAGCGAA		
**OMPA+2 CYP6M7F**	CAAAATGTCTAGCGGCTCCATCGGAGC GGCCTGCGCTACGGTAGCGAA		
**OMPACYP6P9aR**	TCTAGAGAATTCTCACAATTTTTCCACCTTCAAG		
**OMPACYP6P9bR**	TCTAGAGAATTCTTACACCTTTTCTACCTTCAAG		
**OMPACYP6M7R**	TCTAGAGAATTCTCATGTGCTCAGCTTTTCCACC		
**Primers used for amplification of CYP6M7, CYP6P9a and CYP6P9b for polymorphism analysis**			
**CYP6P9a**	ATCCCTAACTATTAAAAGGCAAT	TCACAATTTTTCCACCTTCAAGTAATTACCCGC	2.2
**CYP6P9b**	CATACTCATAATAACTAGACGCG	TTACACCTTTTCTACCTTCAAGTAATTACCCGC	1.8
**CYP6M7**	TCTTGGATTCTTGTAAGAGCGATA	TCATGTGCTCAGCTTTTCCACCTTCAAC	2.2

Green is *EcoRI*, Purple is *NdeI* and Red is *XbaI* site

Table S2: Top 50 probes the most commonly up-regulated in R-S in Mozambique, Malawi and Zambia

ProbeName	Systematic name	Mozambique FC	Malawi FC	Zambia FC	Ortholog in *An. gambiae*	Description
CUST_30_PI406199775	CYP6P9b	88.2	24.0	11.9	AGAP002865-PA	cytochrome p450
CUST_27_PI406199775	CYP6P9a	75.5	39.4	15.6	AGAP002865-PA	cytochrome p450
CUST_25_PI406199775	CYP6P9a	50.6	16.3	8.8	AGAP002865-PA	cytochrome p450
CUST_7369_PI426302897	Afun007369 (CYP6P9b)	30.5	2.5	3.8	AGAP002865-PA	cytochrome p450
CUST_7663_PI426302897	Afun007663 (CYP6M7)	25.7	12.5	37.7	AGAP008213-PA	cytochrome p450
CUST_8239_PI426302897	Afun008239	23.6	18.6	5.7	AGAP002193-PA	Nuclear RNA export factor 1
CUST_26_PI406199775	CYP6P9a	22.2	9.8	5.8	AGAP002865-PA	cytochrome p450
CUST_8887_PI426302897	Afun008887	20.8	8.5	10.0	AGAP011997-PA	nucleotide binding protein 1 (NUBP 1)
CUST_14076_PI426302897	Afun014076	18.6	43.6	7.0	AGAP000603-PA	Hypothetical protein
CUST_4860_PI426302897	Afun004860	16.8	2.8	3.1	NA	NA
CUST_10958_PI426302897	Afun010958	16.2	34.2	14.1	AGAP012235-PA	prefoldin subunit 6
CUST_8614_PI426302897	Afun8416(CYP6AA1)	13.2	5.2	5.3	AGAP002862-PA	cytochrome p450
CUST_11049_PI426302897	Afun011049	11.8	14.8	6.9	AGAP000604-PA	AGAP000604-PA
CUST_9493_PI426302897	Afun009493	11.7	3.4	5.9	AGAP009053-PA	membrane-associated lps-inducible tnf alpha factor protein
CUST_12651_PI426302897	Afun012651	11.7	6.1	5.9	AGAP005769-PA	cg16865 cg16865-pa
CUST_13508_PI426302897	Afun013508	10.1	9.8	3.2	AGAP011951-PA	casps4 protein
CUST_10990_PI426302897	Afun010990	9.8	12.4	15.7	AGAP010693-PA	wd-repeat protein
CUST_10777_PI426302897	Afun010777	9.6	10.0	4.9	AGAP004091-PA	28s ribosomal protein s5
CUST_10150_PI426302897	Afun010150	9.5	4.9	8.5	AGAP011994-PA	phosphopantothenoylcysteine decarboxylase
CUST_6290_PI406199769	combined_c3181	9.1	7.6	10.0		---NA---
CUST_10722_PI426302897	Afun010722	8.5	8.3	5.6	AGAP003551-PA	chitobiosyldiphosphodolichol beta-mannosyltransferase
CUST_2943_PI426302897	Afun002943	8.1	9.5	7.5	AGAP010979-PA	NA

CUST_8968_PI426302897	Afun008968	7.9	8.2	13.9	AGAP011375-PA	selenophosphate synthase
CUST_6291_PI406199769	combined_c3181	7.2	8.6	6.2		---NA---
CUST_15447_PI406199769	combined_c8206	7.1	6.2	4.6		dna ligase 1-like
CUST_1505_PI406199769	combined_c761	6.7	61.6	167.0		---NA---
CUST_1504_PI406199769	combined_c761	6.6	44.3	120.3		---NA---
CUST_15101_PI426302897	Afun015101	5.6	4.0	3.3	AGAP002593-PA	apolipoprotein d
CUST_2806_PI426302897	Afun002806	5.4	9.4	5.8	AGAP012173-PA	AGAP012173-PA
CUST_10591_PI426302897	Afun010591	5.2	4.2	2.2	AGAP005773-PA	gtp-binding protein alpha gnao
CUST_4637_PI406199769	combined_c2347	5.2	4.4	2.6		---NA---
CUST_4636_PI406199769	combined_c2347	5.1	5.0	3.0		---NA---
CUST_3601_PI406199769	combined_c1818	4.8	5.8	5.3		---NA---
CUST_12902_PI406199769	combined_c6589	4.7	17.3	5.7		---NA---
CUST_7584_PI426302897	Afun007584	4.7	5.8	2.8	AGAP012400-PA	alpha-amylase
CUST_2099_PI426302897	Afun002099	4.5	4.1	2.7	NA	NA
CUST_11132_PI426302897	Afun011132	4.5	4.0	2.6	AGAP004286-PA	cg2446- isoform c
CUST_7894_PI426302897	Afun007894	4.5	3.4	2.1	AGAP011477-PA	trypsin delta gamma
CUST_7950_PI426302897	Afun007950	4.4	6.7	5.2	AGAP001045-PA	AGAP001045-PA [Anopheles gambiae
CUST_25_PI426302915	CYP6Y2	4.4	2.9	3.1		cytochrome p450
CUST_7537_PI426302897	Afun007537	4.1	8.2	3.0	AGAP008288-PA	timeless protein
CUST_7469_PI426302897	Afun007469 (CYP9J11)	4.0	3.1	2.6	AGAP012296-PA	cytochrome p450
CUST_4138_PI426302897	Afun004138	4.0	3.1	2.3	AGAP011560-PA	AGAP011560-PA
CUST_12194_PI426302897	Afun012194 (CYP6P2)	3.9	2.7	2.6	AGAP002869-PA	cytochrome p450
CUST_12197_PI426302897	Afun012197 (CYP304B1)	3.9	2.8	2.8	AGAP003066-PA	cytochrome p450
CUST_10982_PI426302897	Afun010982	3.9	2.2	3.5	AGAP003357-PA	AGAP003357-PA
CUST_12569_PI426302897	Afun012569	3.7	2.7	3.3	AGAP008926-PA	spingomyelin synthetase
CUST_4812_PI426302897	Afun004812	3.7	3.6	2.7	NA	NA
CUST_767_PI426302897	Afun000767	3.6	2.9	3.2	AGAP009159-PA	amp dependent ligase

Table S3: Top 20 probes the most commonly down-regulated in R-S in Mozambique, Malawi and Zambia

ProbeName	Systematic name	Mozambique FC	Malawi FC	Zambia FC		Description
CUST_10207_PI426302897	Afun010207	41.5	16.1	7.2	AGAP002850-PA	niemann-pick type c-
CUST_203_PI426302897	Afun000203	35.1	8.3	5.6	AGAP007563-PA	myosin light chain kinase
CUST_11900_PI426302897	Afun011900	32.1	29.9	40.4	AGAP010560-PA	cationic amino acid transporter
CUST_3710_PI406199769	combined_c1873	28.2	29.5	29.0		monkey king protein
CUST_2906_PI406199769	combined_c1465	26.5	19.7	36.7		---NA---
CUST_10329_PI426302897	Afun010329	26.5	26.9	12.7	AGAP005314-PA	lsm6 protein
CUST_11983_PI406199798	AGAP012280-RA	21.4	40.0	9.8	AGAP012280-RA	isoform c
CUST_3711_PI406199769	combined_c1873	19.8	45.4	17.9		monkey king protein
CUST_15960_PI406199769	combined_c8463	19.1	32.7	18.7		---NA---
CUST_8360_PI426302897	Afun008360	18.2	4.1	7.9	AGAP000183-PA	AGAP000183-PA
CUST_14122_PI426302897	Afun014122	15.1	8.4	8.1	AGAP000718-PA	monocarboxylate transporter
CUST_1747_PI406199769	combined_c882	14.5	7.5	8.5		---NA---
CUST_4393_PI426302897	Afun004393	12.8	11.5	12.0	AGAP000718-PA	monocarboxylate transporter
CUST_11294_PI426302897	Afun011294	10.5	7.2	6.3	AGAP006434-PA	peritrophic matrix protein 14
CUST_7771_PI426302897	Afun007771	10.0	6.7	3.2	AGAP000849-PA	nadh dehydrogenase
CUST_1855_PI426302897	Afun001855	9.5	3.5	3.0	NA	NA
CUST_7018_PI406199798	AGAP009859-RA	8.8	13.0	5.8	AGAP009859-RA	AGAP009859-PA
CUST_5526_PI406199769	combined_c2797	8.4	6.5	7.8		---NA---
CUST_11343_PI426302897	Afun011343	8.3	5.9	3.5	AGAP002578-PD	isoform h
CUST_3503_PI406199772	CD577635.1	8.1	3.7	2.9		---NA---

Table S4: Summary statistics for polymorphism of *CYP6M7* between susceptible and resistant mosquitoes in Zambia, Malawi and Mozambique

	Complete sequenced region (2148 bp)								Coding region (1452bp)								Non-coding region (696 bp)				
**Samples**	**N**	**S**	**h**	**Syn**	**NSyn**	**π**	**D**	**D***	**S**	**h**	**π**	**Ka**	**ks**	**Ka/ks**	**D**	**D***	**S**	**h**	**π**	**D**	**D***
**Zambia**																					
**Alive**	10	88	9	40	14	1.6	0.33^{ns}	0.92^{ns}	54	7	1.4	0.41	4.8	0.085	0.42^{ns}	0.86^{ns}	39	9	2.1	0.19^{ns}	0.98^{ns}
**dead**	10	117	10	43	16	2.2	0.61^{ns}	0.87^{ns}	59	10	1.5	0.44	5.03	0.087	0.22^{ns}	0.51^{ns}	62	9	3.9	0.97^{ns}	1.1^{ns}
**All Zambia**	20	138	19	49	20	2.1	0.24^{ns}	0.91^{ns}	69	17	1.45	0.42	4.9	0.085	0.34^{ns}	0.64^{ns}	78	18	3.4	0.13^{ns}	1.3^{ns}
**Malawi**																					
**Alive**	10	89	5	40	8	1.3	-0.65^{ns}	-0.54^{ns}	48	5	0.97	0.21	3.4	0.061	-0.84^{ns}	-0.70^{ns}	46	5	2.2	-0.43^{ns}	-0.35^{ns}
**dead**	8	72	8	20	8	1.45	0.38^{ns}	0.65^{ns}	28	8	0.68	0.22	2.2	0.1	-0.40^{ns}	-0.35^{ns}	47	7	3.07	0.86^{ns}	1.25^{ns}
**All Malawi**	18	104	12	40	12	1.3	-0.3^{ns}	0.03^{ns}	52	11	0.82	0.22	2.8	0.078	-0.87^{ns}	-0.91^{ns}	57	11	2.5	0.22^{ns}	0.73^{ns}
**Mozambique**																					
**All alive**	10	125	10	50	19	2.2	0.26^{ns}	0.51^{ns}	69	10	1.7	0.61	5.6	0.11	0.23^{ns}	0.49^{ns}	60	10	3.3	0.29^{ns}	0.52^{ns}
**All dead**	10	104	10	35	14	1.9	0.42^{ns}	1.05^{ns}	49	9	1.3	0.45	3.9	0.11	0.3^{ns}	1.12^{ns}	57	10	3.2	0.52^{ns}	0.98^{ns}
**All Moz**	20	144	20	52	23	2.1	0.26^{ns}	0.78^{ns}	78	19	1.52	0.54	4.8	0.11	0.04^{ns}	0.71^{ns}	72	20	3.3	0.49^{ns}	0.68^{ns}
**All samples from the three countries**																					
**All alive**	30	157	24	58	23	2.04	0.08^{ns}	0.75^{ns}	84	20	1.55	0.46	5.2	0.088	0.25^{ns}	0.57^{ns}	85	24	3.07	-0.087^{ns}	0.87^{ns}
**All dead**	28	193	28	57	29	2.3	-0.23^{ns}	0.86^{ns}	86	26	1.47	0.44	4.9	0.089	-0.12^{ns}	0.20^{ns}	117	26	4.1	-0.31^{ns}	1.22^{ns}
**Total all**	58	226	51	67	36	2.2	-0.4^{ns}	0.61^{ns}	100	43	1.5	0.45	5.1	0.088	-0.24^{ns}	-0.003^{ns}	126	49	3.7	-0.54^{ns}	1.09^{ns}

N= number of sequences (2n); S, number of polymorphic sites; Syn, Synonymous mutations; Nsyn, Non-synonymous mutations; π, nucleotide diversity; D and D* Tajima's and Fu and Li's statistics; ns, not significant; π, ka and ks are muliplied by 10^2.

Table S5: Codon-based Test of Selection for *CYP6M7* for analysis averaging over all sequence pairs within each group.

	**dN-dS**	**% of significant sequence pairs**
Zambia resistant	5.8***	
Zambia susceptible	5.7***	
Total Zambia	6.0***	96.3
Malawi resistant	5.7***	
Malawi susceptible	4.2***	
Total Malawi	5.3***	64
Mozambique resistant	6.3***	
Mozambique susceptible	5.1***	
Total Mozambique	6.1***	98.4

The probability of rejecting the null hypothesis of strict-neutrality (dN = dS) in favor of the alternative hypothesis (dN < dS) is shown. The test statistic (dN - dS) is shown in the Stat column. dS and dN are the numbers of synonymous and non-synonymous substitutions per site, respectively. The variance of the difference was computed using the bootstrap method (500 replicates).

Table S6: Summary statistics for polymorphism of *CYP6P9a* between susceptible and resistant mosquitoes in Zambia, Malawi and Mozambique

	Complete sequenced region (2053 bp)								Coding region (1527 bp)								Non-coding region (526 bp)				
**Samples**	**N**	**S**	**h**	**Syn**	**NSyn**	**π**	**D**	**D***	**S**	**h**	**π**	**Ka**	**ks**	**Ka/ks**	**D**	**D***	**S**	**h**	**π**	**D**	**D***
									**Zambia**												
**Alive**	10	3	3	0	0	0.04	-0.5^{ns}	0.17^{ns}	0	1	0	0.0	0.0	und	und	und	3	3	0.17	0.50^{ns}	0.17^{ns}
**dead**	10	42	8	12	17	0.6	-0.76^{ns}	-0.87^{ns}	29	7	0.55	0.47	0.73	0.64	-0.99^{ns}	-0.92^{ns}	14	6	0.89	-0.24^{ns}	-0.68^{ns}
**All Zambia**	20	42	10	12	17	0.36	-1.5	-1.77	29	7	0.29	0.27	0.37	0.73	-1.78^{ns}	-1.8^{ns}	14	8	0.59	-0.77^{ns}	-1.39^{ns}
									**Malawi**												
**Alive**	10	2	3	0	1	0.036	0.12^{ns}	-0.28^{ns}	1	2	0.013	0.017	0.0	und	-1.1^{ns}	-1.2^{ns}	1	2	0.10	1.3^{ns}	0.8^{ns}
**dead**	10	14	6	4	3	0.18	-1.22^{ns}	-1.23^{ns}	7	4	0.11	0.064	0.27	0.24	-1.3^{ns}	-1.13^{ns}	7	4	0.36	-0.96^{ns}	-1.13^{ns}
**All Malawi**	20	14	6	4	3	0.11	-1.64^{ns}	-1.75^{ns}	7	4	0.063	0.041	0.14	0.23	-1.67^{ns}	-1.2^{ns}	7	4	0.23	-1.27^{ns}	-1.83^{ns}
									**Mozambique**												
**All alive**	14	4	5	1	2	0.045	-0.84^{ns}	0.3^{ns}	3	4	0.052	0.045	0.075	0.6	-0.49^{ns}	1.07^{ns}	1	2	0.027	-1.15^{ns}	-1.40^{ns}
**All dead**	12	50	9	31	6	0.73	-0.42^{ns}	1.55*	37	6	0.72	0.14	2.7	0.051	-0.43^{ns}	1.49**	13	4	0.75	-0.36^{ns}	1.48**
**All Moz**	26	53	13	32	8	0.38	-1.67^{ns}	1.67**	40	9	0.38	0.094	1.3	0.072	-1.67^{ns}	1.59**	13	4	0.38	-1.4^{ns}	1.49*
									**All samples from the three countries**												
**All alive**	34	8	8	1	3	0.04	-1.5^{ns}	0.01^{ns}	4	5	0.026	0.062	0.033	1.89	-1.46^{ns}	0.0082^{ns}	4	4	0.10	-1.09^{ns}	0.008^{ns}
**All dead**	32	91	20	39	25	0.57	-1.87*	-0.87^{ns}	64	14	0.51	0.25	1.36	0.18	-1.9*	0.16^{ns}	30	10	0.77	-1.63^{ns}	-0.75^{ns}
**Total all**	66	94	25	40	27	0.31	-2.3**	-0.57^{ns}	67	17	0.26	0.138	0.69	0.2	-2.4**	-0.1^{ns}	30	12	0.44	-2.03*	-1.35^{ns}

N= number of sequences (2n); S, number of polymorphic sites; Syn, Synonymous mutations; Nsyn, Non-synonymous mutations; π, nucleotide diversity; D and D* Tajima's and Fu and Li's statistics; ns, not significant; π, ka and ks are muliplied by 10^2.

Table S7: Summary statistics for polymorphism of *CYP6P9b* between susceptible and resistant mosquitoes in Zambia, Malawi and Mozambique

	Complete sequenced region (1812bp)								Coding region (1527 bp)								Non-coding region (285 bp)				
**Samples**	**N**	**S**	**h**	**Syn**	**NSyn**	**π**	**D**	**D***	**S**	**h**	**π**	**ka**	**ks**	**Ka/ks**	**D**	**D***	**S**	**h**	**π**	**D**	**D***
**Zambia**																					
**Alive**	10	2	3	1	1	0.042	0.22^{ns}	-0.28^{ns}	2	3	0.049	0.047	0.057	0.82	0.22^{ns}	-0.28^{ns}	0	1	0.0	und	und
**dead**	10	23	6	14	6	0.29	-1.65^{ns}	-1.90^{ns}	20	6	0.31	0.15	0.88	0.17	-1.58^{ns}	-1.81^{ns}	3	3	0.21	-1.56^{ns}	-1.78^{ns}
**All Zambia**	20	24	7	15	6	0.16	-2.1*	-3.15**	21	7	0.18	0.10	0.44	0.23	-2.03*	-3.0**	3	3	0.10	-1.72^{ns}	-2.38^{ns}
**Malawi**																					
**Alive**	10	0	1	0	0	0	und	und	0	1	0	0	0	und	und	und	0	1	0.0	und	und
**dead**	10	12	2	6	3	0.13	-1.96*	-2.3*	9	2	0.12	0.051	0.34	0.15	-1.9*	-2.22**	3	2	0.21	1.56^{ns}	1.78^{ns}
**All Malawi**	20	12	2	6	3	0.066	-2.31**	-3.4*	9	2	0.059	0.026	0.17	0.15	-2.22**	-3.27**	3	2	0.10	-1.72^{ns}	-2.38^{ns}
**Mozambique**																					
**All alive**	14	1	2	0	1	0.015	-0.34^{ns}	-0.71^{ns}	1	2	0.017	0.022	0	und	-0.34^{ns}	0.71^{ns}	0	1	0.0	und	und
**All dead**	12	43	3	36	3	0.68	-0.6^{ns}	1.1^{ns}	39	3	0.73	0.103	2.8	0.036	-0.62^{ns}	1.04^{ns}	4	2	0.43	-0.29	1.19
**All Moz**	26	44	4	36	4	0.34	-1.77^{ns}	1.03^{ns}	40	4	0.36	0.063	1.36	0.046	-1.78^{ns}	0.95^{ns}	4	2	0.21	-1.16^{ns}	1.07^{ns}
**All samples from the three countries**																					
**All alive**	34	4	5	2	2	0.047	-0.3^{ns}	-0.008^{ns}	4	5	0.056	0.032	0.14	0.23	-0.03^{ns}	-0.008^{ns}	0	1	0.0	und	und
**All dead**	32	60	10	39	10	0.42	-1.85*	-0.04^{ns}	52	10	0.44	0.11	1.56	0.07	-1.73^{ns}	0.32^{ns}	9	5	0.3	-1.9*	-1.6^{ns}
**Total all**	66	62	12	40	11	0.23	-2.3**	-0.56	54	12	0.25	0.069	0.84	0.08	-2.23**	-0.08^{ns}	9	5	0.15	-2.07*	-2.15^{ns}

N= number of sequences (2n); S, number of polymorphic sites; Syn, Synonymous mutations; Nsyn, Non-synonymous mutations; π, nucleotide diversity; D and D* Tajima's and Fu and Li's statistics; ns, not significant; π, ka and ks are muliplied by 10^2.

Table S8: Genetic differentiation using K_{ST} for *CYP6P9a, CYP6P9b* and *CYP6M7*

CYP6P9a			CYP6P9b			CYP6M7		
	Mozambique	Zambia		Mozambique	Zambia		Mozambique	Zambia
Malawi	0.021^{ns}	0.058***	Malawi	0.012^{ns}	0.217***	Malawi	0.177***	0.073***
Mozambique		0.04***	Mozambique		0.097***	Mozambique		0.106***

PERMTEST calculates Hudson's K_{ST} statistic of genetic differentiation. K_{ST} is equal to 12KS/KT, where KS is a weighted mean of K1 and K2 (mean number of differences between sequences in subpopulations 1 and 2, respectively) and KT represents the mean number of differences between two sequences regardless of their subpopulation. The null hypothesis of no genetic differentiation will be rejected (P<0.05) when KS is small and K_{ST} is close to 1.PM test; Probability obtained by the permutation test with 1000 replicates); ns, not significant; *, 0.01<P<0.05; **, 0.001<P<0.01; ***, P<0.001
