# Supplementary material for: The highly polymorphic CYP6M7 cytochrome P450 gene partners with the directionally selected CYP6P9a and CYP6P9b genes to expand the pyrethroid resistance front in the malaria vector Anopheles funestus in Africa
Source: BMC Genomics. 2014 Sep 27;15(1):817. doi: 10.1186/1471-2164-15-817 (PMC4192331; doi:10.1186/1471-2164-15-817)
Supplement: Supplementary file 2 — Additional file 2: Figure S1: Gene expression analysis. Figure S2. Maximum likelihood (ML) tree of full-length CYP6M7 cDNA haplotypes from various regions of Africa. Figure S3. Analysis of polymorphisms of CYP6M7, CYP6P9a and CYP6P9b. Figure S4. Protein variants among resistant and susceptible mosquitoes from all three countries. Figure S5. Phylogenetic analysis of CYP6M7. Figure S6. Impact of permethrin resistance on genetic diversity. Figure S7. Comparative analysis of the haplotypes of the three genes based only on coding regions among susceptible and resistant mosquitoes from the three countries using a 95% parsimony network. (PDF 638 KB) [file 12864_2014_6509_MOESM2_ESM.pdf]

Supplementary figures

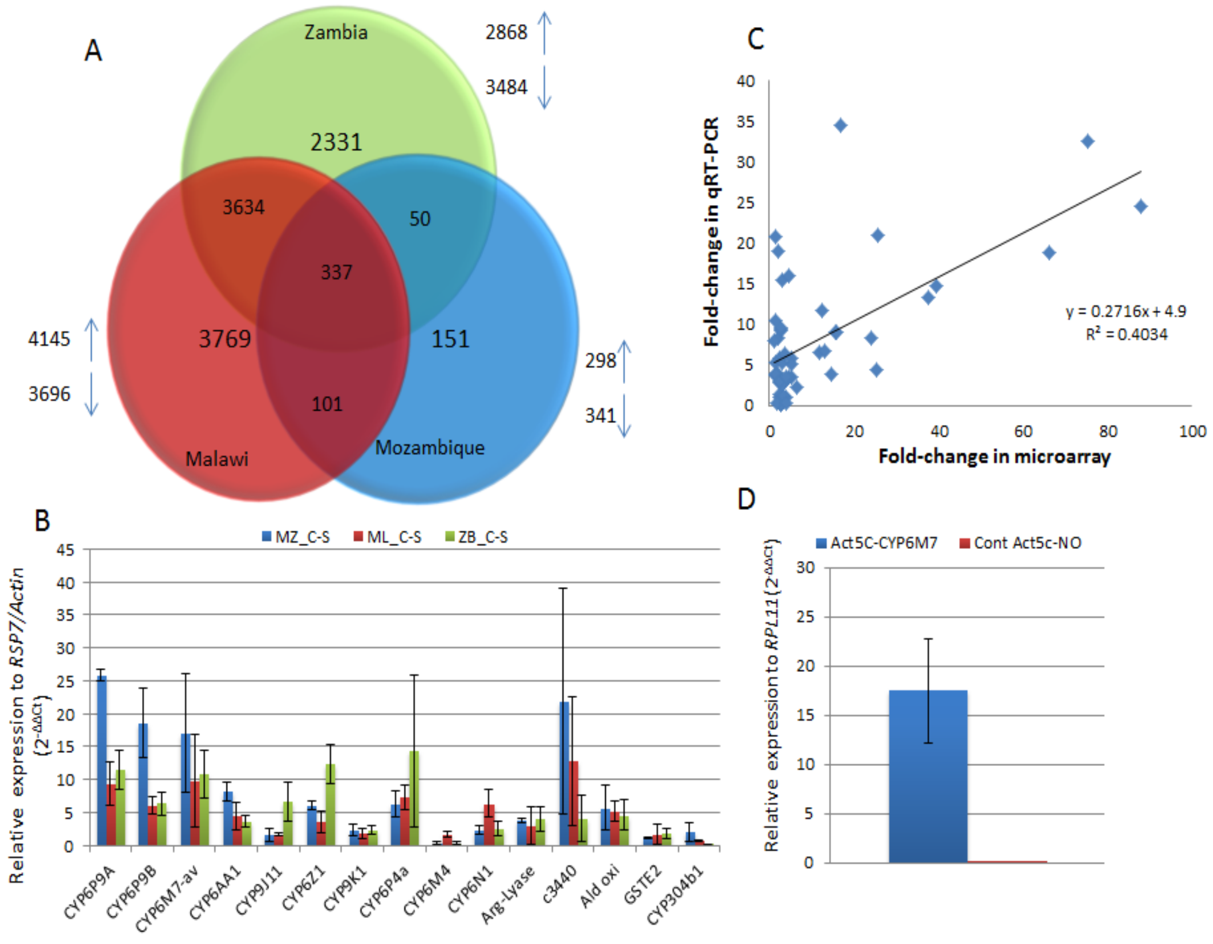

Figure S1: Gene expression analysis. A) Summary of probes differentially regulated in each of the samples from the 3 countries at P<0.05 with FC>2 up- or down-regulation. B) Differential expression of 15 genes up-regulated between control non-exposed (C) and FANG-susceptible (S) mosquitoes from Mozambique (MZ), Malawi (ML) and Zambia (ZB), as identified by microarray analysis. C) Correlation between the microarray and qRT-PCR data for genes selected from the list of up-regulated probes. D) Relative expression of the transgene CYP6M7 in the transgenic *D. melanogaster* strain (Act5C-CYP6M7) and the control sample with no *CYP6M7* expression (Cont Act5c-No). The data shown are the mean ± SEM (n= 3).

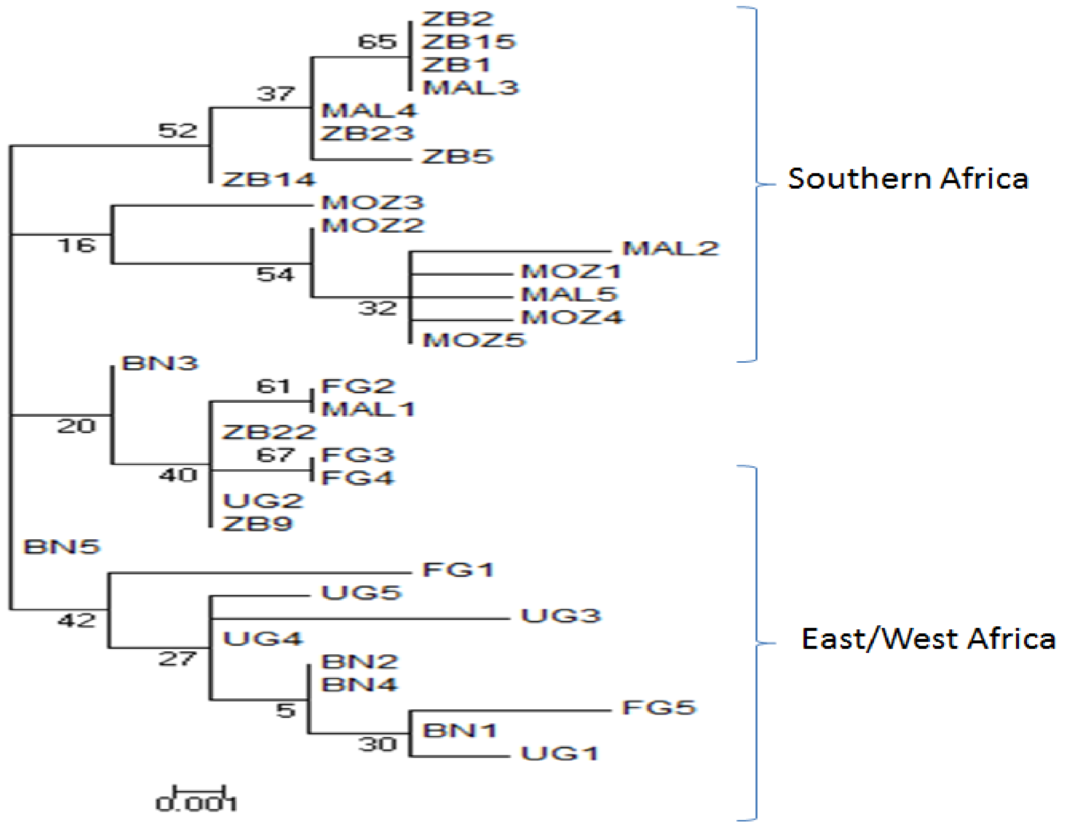

Figure S2: Maximum likelihood (ML) tree of full-length *CYP6M7* cDNA haplotypes from various regions of Africa. It shows a clade of haplotypes from the Southern African countries of Mozambique (MOZ), Malawi (MAL) and Zambia (ZB). Another clade comprises haplotypes from Uganda (UG) in East Africa, Benin (BN) in West Africa and the fully susceptible laboratory strain FANG (FG), which is originally from Angola.

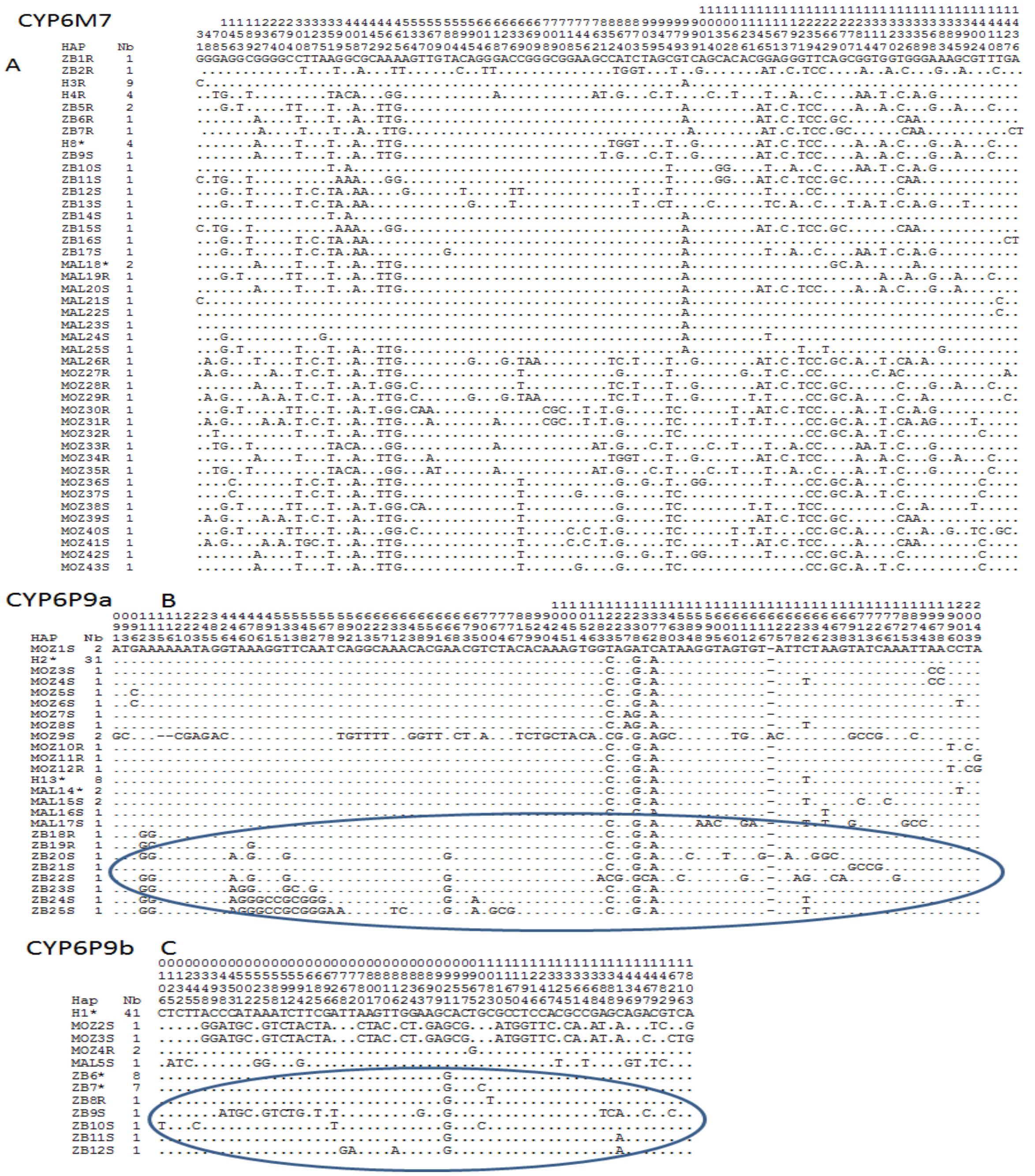

Figure S3: Analysis of polymorphisms of *CYP6M7, CYP6P9a* and *CYP6P9b*. A) Schematic representation of *CYP6M7* haplotypes for the coding region among resistant and susceptible mosquitoes from all three countries. The polymorphic positions are indicated by numbers above the nucleotide, and the haplotypes are named with the initial of the country in which it is predominant: ZB for Zambia, MAL for Malawi and MOZ for Mozambique. Haplotypes named with H are found in more than one country. R, S, or * are added to the names when the haplotype is found in resistant or susceptible mosquitoes, respectively. B) and C) represent the full length (coding and non-coding regions) of *CYP6P9a* and *CYP6P9b* haplotypes, respectively with the polymorphic positions specific to Zambia encircled to highlight the higher variability and differences observed for both genes in Zambia compared to Malawi and Mozambique.

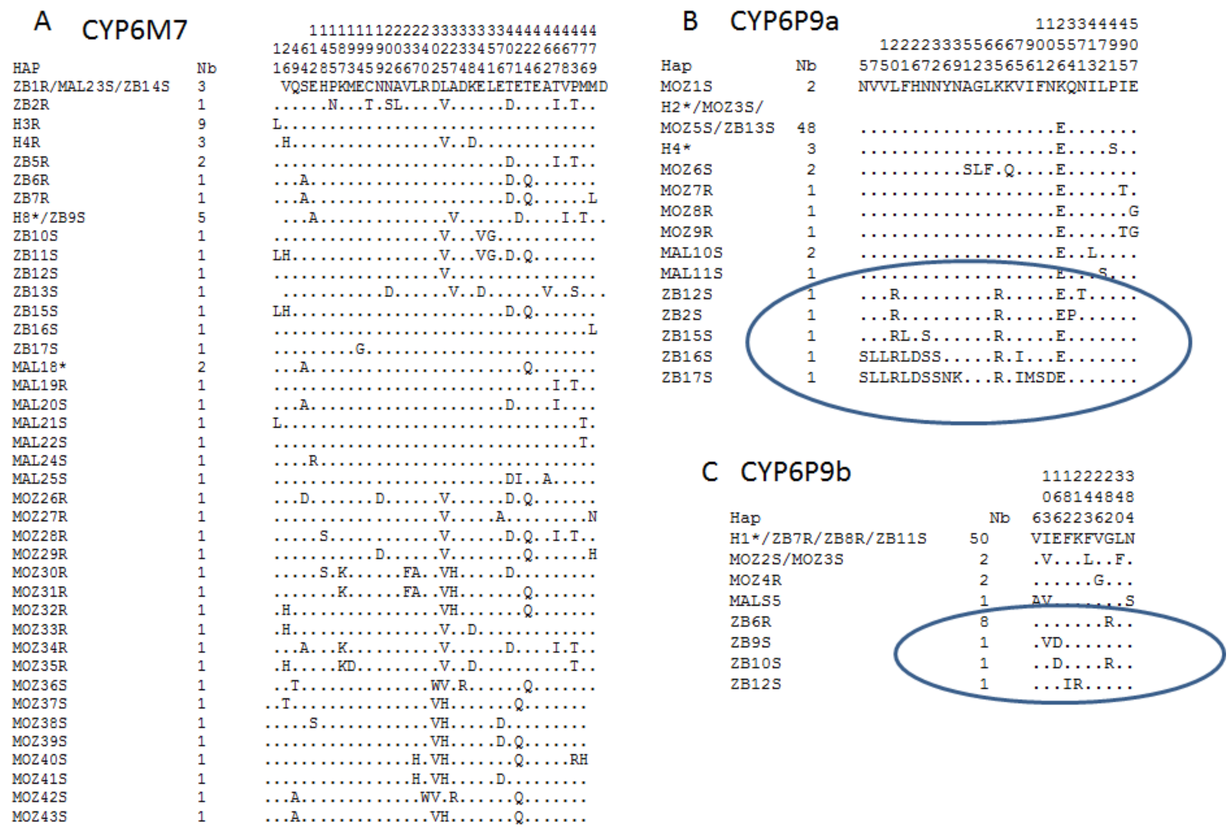

Figure S4: **Protein variants among resistant and susceptible mosquitoes from all three countries.** A) Schematic representation of amino acid polymorphic positions in *CYP6M7* haplotypes in resistant and susceptible mosquitoes from all three countries. B) and C) represent *CYP6P9a* and *CYP6P9b* haplotypes, respectively. For *CYP6P9a* and *CYP6P9b*, the polymorphic positions specific to Zambia are encircled to highlight the higher variability and differences observed for both genes in Zambia compared Malawi and Mozambique.

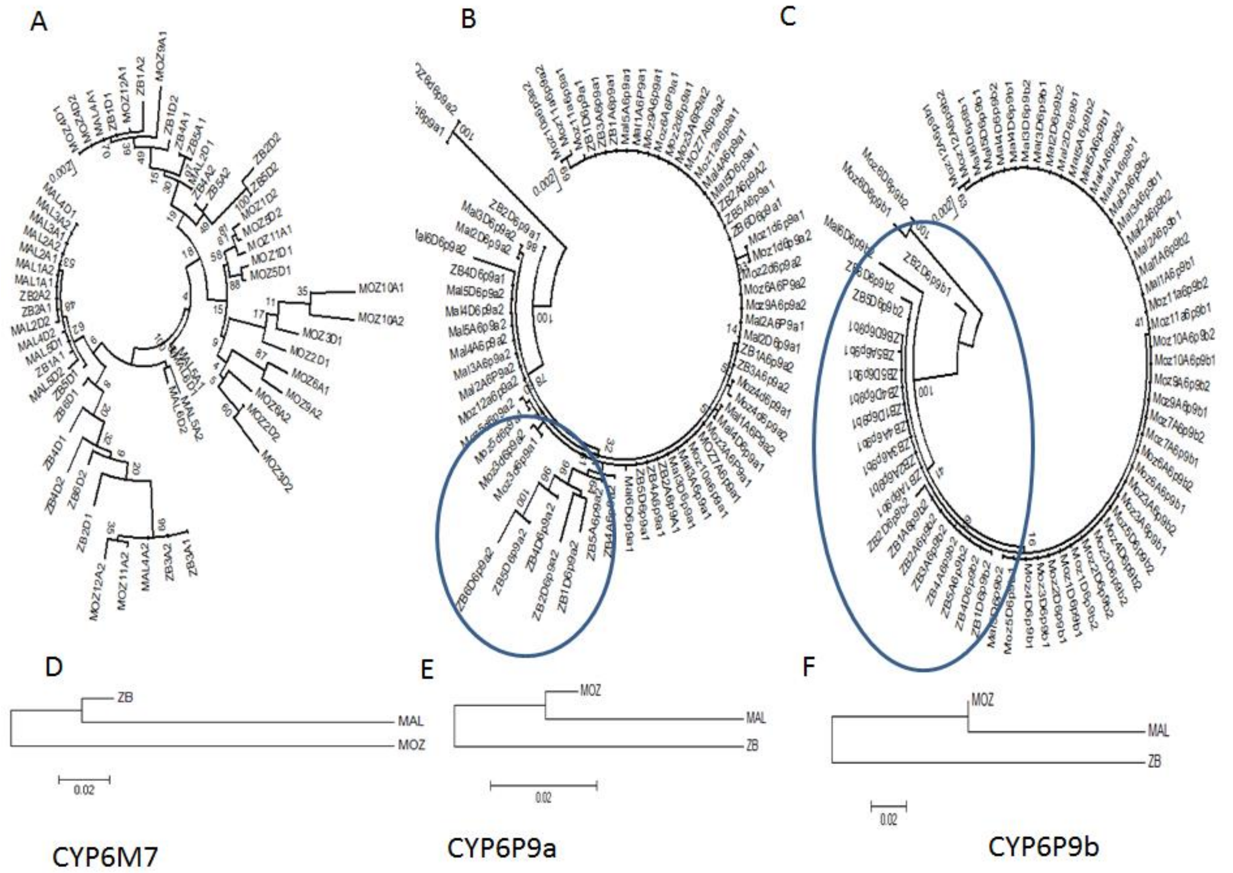

Figure S5: **Phylogenetic analysis of *CYP6M7*.** Maximum likelihood (ML) tree of *CYP6M7* haplotypes between permethrin-resistant (A for alive after exposure) and susceptible (D for dead after exposure) mosquitoes. The tree with the highest log likelihood (-4115.4346) is shown. Initial tree(s) for the heuristic search were obtained automatically by applying Neighbor-Joining and BioNJ algorithms to a matrix of pairwise distances estimated using the Maximum Composite Likelihood (MCL) approach and then selecting the topology with a superior log likelihood value. The tree is drawn to scale, with branch lengths measured in the number of substitutions per site. The analysis involved 58 nucleotide sequences from Mozambique (MOZ), Malawi (MAL) and Zambia (ZB). Haplotypes ending with 1 or 2 represent the two haplotypes of each mosquito. For *CYP6P9a* and *CYP6P9b*, a cluster of haplotypes from Zambia is observed and is circled in blue. Neigbhor-joining trees based on genetic distances between Malawi (MAL), Mozambique (MOZ) and Zambia (ZB) populations (*Nst* estimates) for *CYP6M7* (D), *CYP6P9a* (E) and *CYP6P9b* (F).

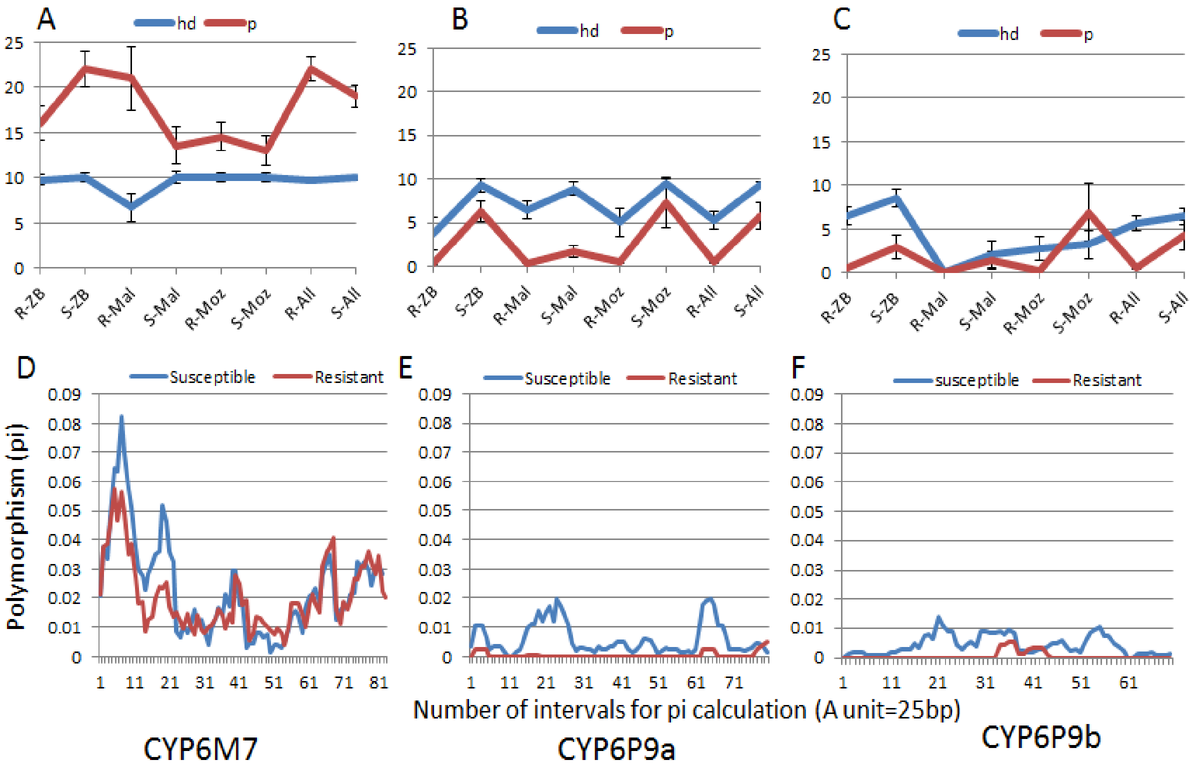

Figure S6: **Impact of permethrin resistance on genetic diversity**. A) Variations in the genetic parameters of *CYP6M7* between permethrin-susceptible (S) and -resistant (R) mosquitoes from Zambia (ZB), Malawi (Mal) and Mozambique (Moz) and combined samples from all three countries. The genetic diversity (π) was multiplied by 10^3, and haplotype diversity (hd) was multiplied by 10. B) and C) show identical analyses of *CYP6P9a* and *CYP6P9b*, respectively. D) Sliding-window analysis of polymorphism (π) of *CYP6M7* between all permethrin-susceptible and -resistant mosquitoes from the three countries, with π calculated at 25 bp intervals along the gene. E) and F) show identical analyses of *CYP6P9a* and *CYP6P9b*, respectively.

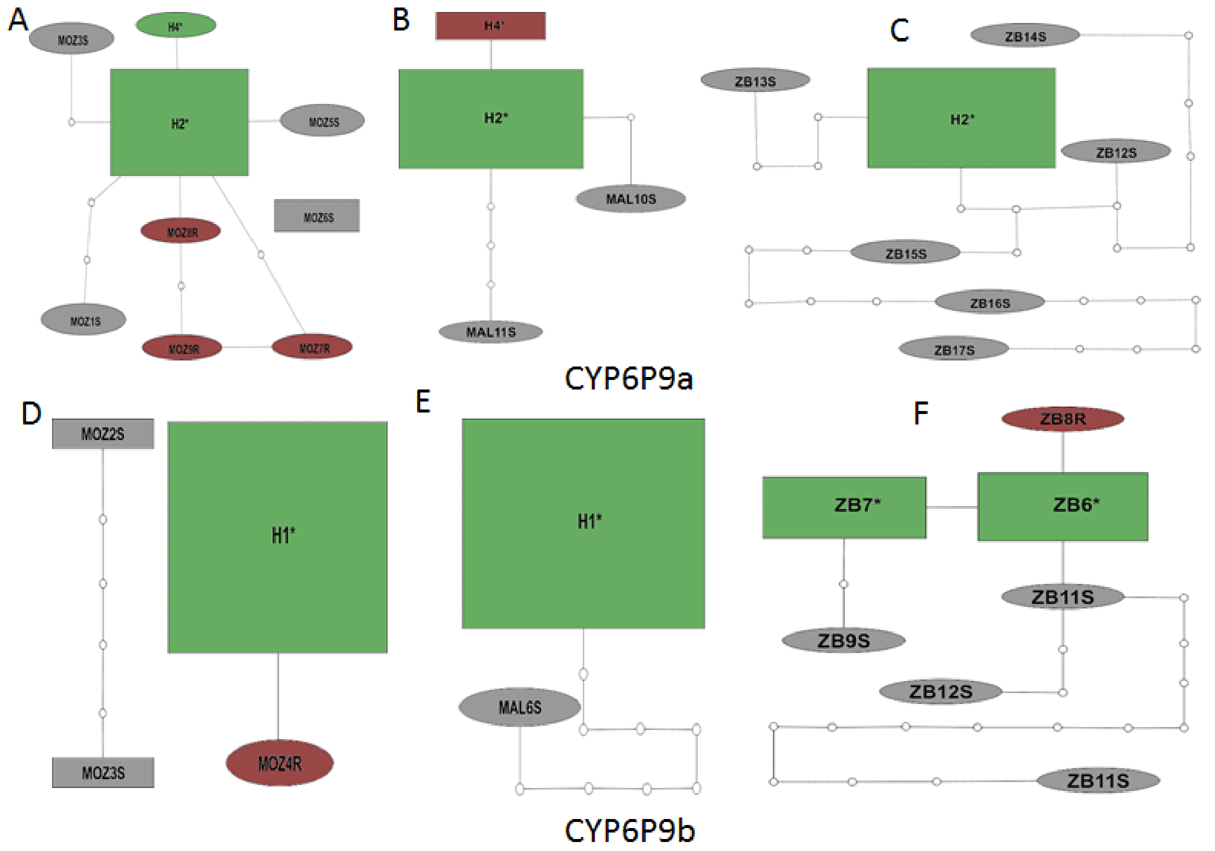

Figure S7: **Comparative analysis of the haplotypes of the three genes based only on coding regions among susceptible and resistant mosquitoes from the three countries using a 95% parsimony network.** A), B) and C) represent the analysis of *CYP6P9a* in the Mozambican, Malawian and Zambian samples, respectively. Haplotypes are represented as an oval or a rectangle scaled to reflect their frequencies. Lines connecting haplotypes and each node represent a single mutational event. Gray shapes represent haplotypes unique in susceptible mosquitoes; green shapes represent haplotypes predominantly found in resistant mosquitoes as well as some dead mosquitoes; red shapes represent haplotypes unique to resistant mosquitoes. Some haplotypes with >20 mutation differences from others could not be linked to the major network. D), E) and F) represent the analysis of *CYP6P9b* in the Mozambican, Malawian and Zambian samples, respectively.
